# Cell-matrix adhesion and cell-cell adhesion differentially control basal myosin oscillation and *Drosophila* egg chamber elongation

**DOI:** 10.1038/ncomms14708

**Published:** 2017-04-13

**Authors:** Xiang Qin, Byung Ouk Park, Jiaying Liu, Bing Chen, Valerie Choesmel-Cadamuro, Karine Belguise, Won Do Heo, Xiaobo Wang

**Affiliations:** 1 LBCMCP, Centre de Biologie Intégrative (CBI), Université de Toulouse, CNRS, UPS, Toulouse 31062, France; 2 Center for Cognition and Sociality, Institute for Basic Science (IBS), Daejeon 34141, Republic of Korea; 3 Department of Anesthesia, Southwest Hospital, Third Military Medical University, Chongqing 400038, China; 4 Department of Biological Sciences, Korea Advanced Institute of Science and Technology (KAIST), Daejeon 34141, Republic of Korea

## Abstract

Pulsatile actomyosin contractility, important in tissue morphogenesis, has been studied mainly in apical but less in basal domains. Basal myosin oscillation underlying egg chamber elongation is regulated by both cell–matrix and cell–cell adhesions. However, the mechanism by which these two adhesions govern basal myosin oscillation and tissue elongation is unknown. Here we demonstrate that cell–matrix adhesion positively regulates basal junctional Rho1 activity and medio-basal ROCK and myosin activities, thus strongly controlling tissue elongation. Differently, cell–cell adhesion governs basal myosin oscillation through controlling medio-basal distributions of both ROCK and myosin signals, which are related to the spatial limitations of cell–matrix adhesion and stress fibres. Contrary to cell–matrix adhesion, cell–cell adhesion weakly affects tissue elongation. *In vivo* optogenetic protein inhibition spatiotemporally confirms the different effects of these two adhesions on basal myosin oscillation. This study highlights the activity and distribution controls of basal myosin contractility mediated by cell–matrix and cell–cell adhesions, respectively, during tissue morphogenesis.

## Abstract (web summary)

Pulsatile actomyosin contractility during tissue morphogenesis has been mainly studied in apical domains but less is known about the contribution of the basal domain. Here the authors show differential influence of cell-matrix and cell-cell adhesions in regulating oscillations and tissue elongation.

Tissue morphogenesis is an event during which cells undergo dynamic shape changes and remodelling for the acquisition of tissue shape and the maintenance of tissue homeostasis during development^1^,^2^. Tissue elongation is a type of morphogenesis known to be controlled by various mechanisms, including oriented cell division, migration and rearrangement^3^,^4^,^5^,^6^. A newly established model to study tissue elongation is the *Drosophila* ovary^7^, which contains 15 strings of the egg chambers during different developing stages from S1 to S14. The egg chamber is a structure composed of a monolayer follicular epithelium surrounding 16-germline cysts. During oogenesis, the egg chamber gradually changes its shape from round to elongated anterior-posteriorly^7^. This tissue elongation mainly occurs between S5 and S10B, and it is controlled by two distinct phenomena. The first control is the egg chamber global rotation^8^, which facilitates to build up a ‘molecular corset' of the dorsal–ventral (DV) organized extracellular matrix and to favour growth along the anterior-posterior (AP) axis from S5 to S8. The second control is basal myosin oscillation^9^. From early S9 to S10B, non-muscle myosin II (MyoII) will load on the DV polarized basal actin filament and do periodic basal contraction to eventually shape the mature egg.

MyoII oscillation also occurs in apical cell contraction which governs a few types of tissue morphogenesis during *Drosophila* embryo development, such as the invagination of mesoderm cells during gastrulation, the intercalation of ectoderm cells during ventral-lateral elongation, and the periodic contraction of aminoserosa during dorsal closure^10^,^11^,^12^,^13^. Previous studies demonstrated that the small GTPase Rho1 and its downstream kinase ROCK are spatiotemporally correlated with and critically important for apical MyoII oscillation and cell contraction^14^,^15^,^16^,^17^,^18^,^19^. About the upstream control of Rho-ROCK signalling pathway, several studies^20^,^21^,^22^,^23^,^24^ reported that G-protein-coupled-receptors and heterotrimeric G proteins control the RhoGEF2 and Rho1-ROCK pathway, and these controls recruit, activate and polarize the medio-apical and/or junctional MyoII in the invaginating mesoderm cells and the intercalating ectoderm cells.

Concerning the control of basal MyoII oscillation, Rho1 and ROCK have also been shown to regulate basal MyoII intensity in follicle cells^9^ while their spatiotemporal signal dynamics are unknown. In addition, MyoII and F-actin intensities at basal domain of follicle cells are strongly downregulated by the inhibition of cell–cell adhesion or cell–matrix adhesion^9^. However, the controlling mechanism of basal MyoII oscillation by these two types of adhesions is completely unknown. The question is whether both controls are dependent on the Rho-ROCK signalling pathway, actin cytoskeleton network or other factors. Moreover, it is unclear whether the inhibition of cell–cell adhesion might affect the amplitude and period of basal cell contractions and tissue elongation. To clearly determine how these two types of adhesions govern basal MyoII oscillation and the underlying tissue elongation, we need to characterize the Rho-ROCK signalling pathway and actin network in control of basal MyoII oscillation, and the correlation of both factors with cell–cell and cell–matrix adhesions.

Here we report that Integrin-dependent cell–matrix adhesion, but not E-cadherin-dependent cell–cell adhesion, is spatiotemporally correlated with basal MyoII signal. Cell–matrix adhesion positively regulates Rho1 activity at and near basal junction, and also positively controls ROCK and MyoII oscillations at medio-basal region (the middle of basal cortical region). Therefore, cell–matrix adhesion functions as positive control of Rho1-ROCK signalling pathway. Different from cell–matrix adhesion, cell–cell adhesion does not regulate basal Rho1 activity, but controls the subcellular distribution of ROCK and MyoII oscillations via the spatial limitation of actin filament that will load MyoII within medio-basal region. Inhibition of cell–cell adhesion leads to the redistribution of stress fibres from medio-basal region to basal junction and to the abnormal localization of cell–matrix adhesion, which thus affects the localization of basal MyoII oscillation. This difference results in the varied effects on basal MyoII oscillation and morphogenetic tissue elongation.

## Results

### Cell–matrix adhesion correlates with basal MyoII signal

**Figure 1:**
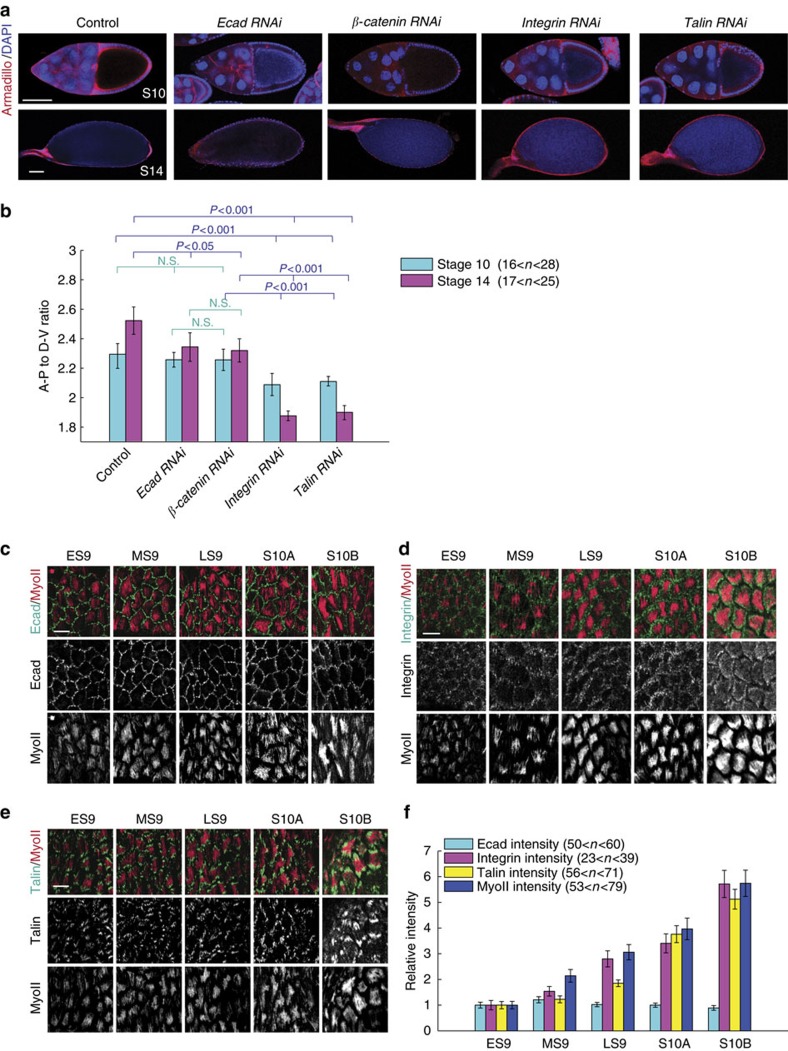
Cell–cell adhesion and cell–matrix adhesion differently correlate with basal MyoII signal and differently regulate organ shape. (**a**) Morphology of stage-10 and stage-14 egg chambers expressing the indicated transgenes, staining by Armadillo and DAPI, 4',6-diamidino-2-phenylindole. Both scale bars are 50 μm. (**b**) Quantification of the A-P to D-V length ratio in S10 and S14 egg chambers with the indicated genetic backgrounds. *n* is the number of samples analysed. Error bars indicate ±s.d. N.S. means no significant difference, while *P*<0.05 and *P*<0.001 mean weak and significant difference by student's *t*-test. (**c**–**e**) Basal views of follicle cells from egg chambers at early stage 9 (ES9), middle stage 9 (MS9), late stage 9 (LS9), stage 10A (S10A) and stage 10B (S10B), marked by the presence of MyoII-mCherry (red) with E-cadherin-GFP (green) (**c**), MyoII-mCherry (red) with β-Integrin-GFP (green) (**d**), and MyoII-mCherry (red) with Talin-GFP (green) (**e**), respectively. All scale bars are 10 μm. (**f**) Quantification of relative basal E-cadherin, β-Integrin, Talin and MyoII intensities at different stages. *n* is the number of samples analysed. Error bars indicate ±s.d.

Basal pulsatile MyoII contractility has been known to control the egg chamber elongation during *Drosophila* oogenesis^9^. Therefore, we tested the effects of cell–matrix and cell–cell adhesions on tissue elongation by the image analysis of egg chamber shapes at S10 and S14, as described previously^9^. Compared with the control egg chamber, inhibition of cell–matrix adhesion by the expression of either *β-Integrin RNAi* or *Talin RNAi* significantly results in the rounder egg chambers at both stages (Fig. 1a,b), which is consistent with our previous observation^9^. However, inhibition of cell–cell adhesion by either *E-cadherin RNAi* or *β-catenin RNAi* expression has very weak effect on the underlying tissue elongation (Fig. 1a,b). We confirmed that the different effects of cell–matrix and cell–cell adhesions on tissue elongation are not due to the knockdown efficiency of *β-Integrin RNAi*, *Talin RNAi* and *E-cadherin RNAi* (Supplementary Fig. 1). Since the tissue shape at S10 might also be controlled by the egg chamber global rotation^8^, we assessed the effect of cell-matrix and cell–cell adhesions on global tissue rotation. Inhibition of cell–matrix and cell–cell adhesions by the respective RNA interference (RNAi) expression does not significantly affect the rotational speed of egg chambers at S6 and S7, compared with control (Supplementary Fig. 2). These results excluded the possibility that the S10 tissue shapes affected by cell–matrix and cell–cell adhesions are dependent on the egg chamber global rotation. Thus, the different effects on tissue elongation indicate that cell–matrix and cell–cell adhesions might differently control basal MyoII oscillation.

Next, we assessed the correlation between adhesions and basal MyoII signal. We compared the endogenous protein levels and the distribution patterns of these two adhesions (green fluorescent protein (GFP)-knockin *E-cadherin, β-Integrin and Talin*^25^,^26^) with those of MyoII at basal domain of follicle cells during the period of basal MyoII oscillation. From early S9 to S10B, basal E-cadherin protein levels have no significant change while β-Integrin and Talin protein levels gradually increase, which correlates with the augmentation of basal MyoII intensity (Fig. 1c–f). In addition, basal E-cadherin is uniformly distributed along the membrane as a punctate structure (Fig. 1c), while β-Integrin and Talin are mainly localized at both sides of basal MyoII fibres aligned at the DV axis (Fig. 1d,e). These results demonstrated that the expression and distribution patterns of β-Integrin and Talin, but not E-cadherin, are correlated with those of basal MyoII signal, suggesting that cell–matrix adhesion might be a positive regulator of basal MyoII oscillation.

Consistent with this correlation, we observed that basal MyoII oscillation can be strongly affected by the expression of various β-Integrin mutants. Cells expressing wild-type (WT) β-Integrin present a normal period of basal MyoII oscillation (Supplementary Fig. 3). However, the expression of mutants (804*stop, N840A and N828A (refs 27, 28)) inhibiting β-Integrin activity strongly decreases basal MyoII intensity and oscillation period (Supplementary Fig. 3). Oppositely, the expression of mutants (YY>FF, G792N and L796R (ref. 28)) enhancing β-Integrin activity significantly increases basal MyoII intensity and oscillation period (Supplementary Fig. 3). Our next question is how cell–matrix adhesion positively controls basal MyoII oscillation.

### Cell–matrix adhesion controls basal Rho1-MyoII activities

**Figure 2:**
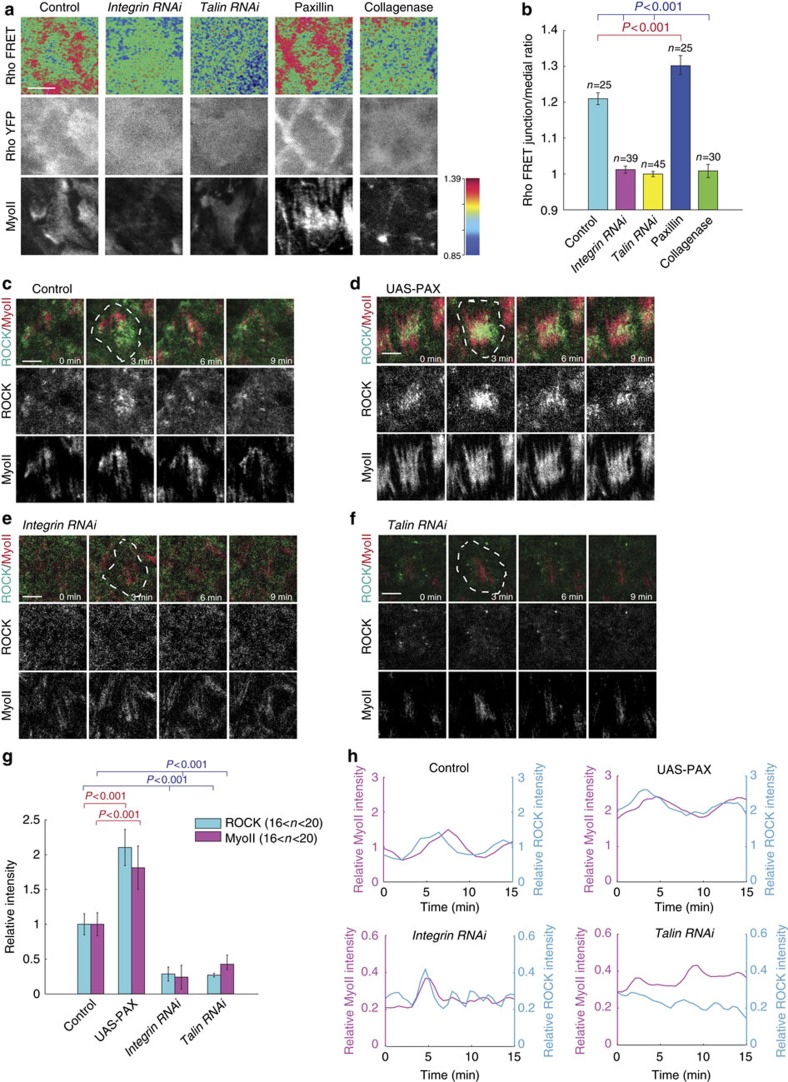
Cell–matrix adhesion positively controls the activity of Rho1 to MyoII signal cascade. (**a**) Representative Rho FRET images, together with YFP channel only and MyoII-mCherry, in the wild type, *β-Integrin RNAi*-expressing, *Talin RNAi*-expressing, Paxillin-expressing follicle cells and the follicle cells with the treatment of collagenase. Top, processed Rho FRET signal; middle, YFP channel only; bottom, MyoII signal. (**b**) Quantification of the basal junction/medio-basal Rho FRET ratio in all indicated conditions. (**c**–**f**) Time-lapse series of one representative wild type (**c**), Paxillin-expressing (**d**), *β-Integrin RNAi*-expressing (**e**) and *Talin RNAi-expressing* (**f**) follicle cell (one cell is marked by dotted line), labelled with ROCK-GFP and MyoII-mCherry. All scale bars are 5 μm. (**g**) Quantification of relative ROCK and MyoII intensities in all indicated conditions. Average value of ROCK and MyoII intensities in one oscillating cycle has been used as one sample for average quantification. *n* is the number of samples analysed. All error bars indicate ±s.d., *P*<0.001 means significant difference by student's *t*-test. (**h**) Quantifications of the dynamic changes of ROCK-GFP and MyoII-mCherry intensities in one representative oscillating cell with control, Paxillin-expressing, *β-Integrin RNAi*-expressing and *Talin RNAi*-expressing conditions, respectively. In (**h**), the intensities of ROCK and MyoII signals in Paxillin-expressing (**d**), *β-Integrin RNAi*-expressing (**e**) and *Talin RNAi*-expressing (**f**) follicle cells have been normalized to those in control cells (**c**).

Rho1 and ROCK have been described to be the major control of pulsatile MyoII contractility in different morphogenetic processes^9^,^14^,^15^,^16^,^17^,^18^,^19^. Thus, we next assessed the correlation between cell–matrix adhesion and basal Rho1-ROCK signals. Rho1 activity, detected by a Rho FRET biosensor^29^ whose development and feasibility have been validated in this study (Supplementary Fig. 4a–c), is mainly enriched at and near basal junction of follicle cells during S9 and S10 (Fig. 2a,b). Moreover, basal junctional Rho1 activity shows some moderate planar cell polarity enriched at the DV axis (Fig. 4c). The basal junctional distribution of Rho1 activity is similar to the basal junctional localization of RhoGEF2 and Rho1 signals (Supplementary Fig. 4d,e). Surprisingly, ROCK and MyoII signals are mainly distributed at the middle region of basal cortex (Fig. 2c and Supplementary Fig. 4d,e). Inhibition of β-Integrin or Talin by RNAi or the treatment with collagenase almost completely abolishes Rho1 activity at and near basal junction, which is consistent with the inhibition of Rho1 activity by the genetic or chemical perturbation; while the enhancement of cell–matrix adhesion by the overexpression of downstream effector Paxillin^30^ increases Rho1 activity at and near basal junction, similar to the effect of RhoGEF2 overexpression (Fig. 2a,b and Supplementary Fig. 4a–c). Consistent with these results, cell–matrix adhesion also strongly governs the intensity and oscillation of ROCK and MyoII signals since the β-Integrin or Talin inhibition by RNAi prominently reduces the intensity and oscillation of both signals at basal domain during S9 and S10 (Fig. 2c,e,f–h and Supplementary Movies 1–3). In the opposite case, the Paxillin overexpression significantly enhances the intensity and oscillation of both signals (Fig. 2d,g,h and Supplementary Movie 4). Altogether, our data demonstrated that cell–matrix adhesion functions as the main positive regulator of basal Rho1-MyoII signal cascade.

**Figure 3:**
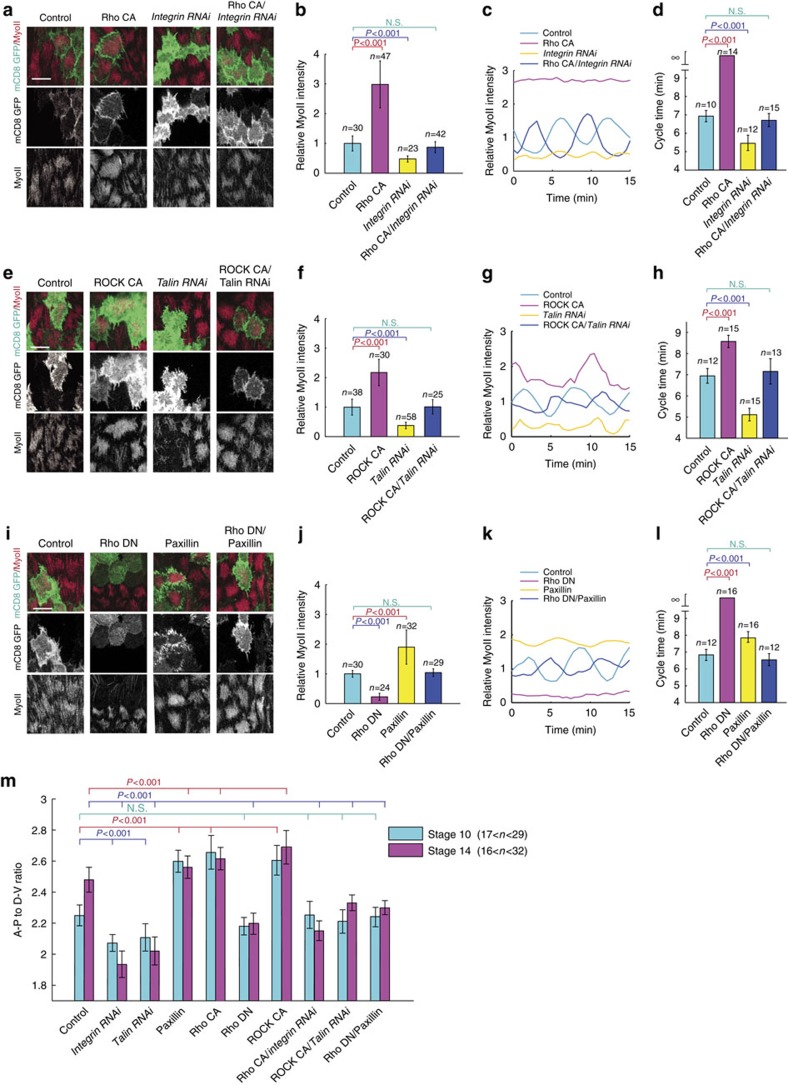
Cell–matrix adhesion regulates basal MyoII oscillation via Rho1 and ROCK signals. (**a**,**e**,**i**) Basal views of follicle cell clones expressing the indicated transgenes, marked by coexpression of mCD8GFP. All scale bars are 10 μm. (**b**,**f**,**j**) Quantifications of relative MyoII intensity in the indicated transgene-expressing GFP-positive cells compared with the GFP-negative wild-type cells in the same sample. (**c**,**g**,**k**) Quantifications of the dynamic changes of MyoII-mCherry intensity in one representative oscillating cell with the indicated genetic backgrounds. Average intensity of MyoII signal in the control oscillating cells is set as 1 in (**c**,**g**,**k**). (**d**,**h**,**l**) Quantifications of average oscillating cycle time period in the indicated genetic backgrounds. Symbol ∞ means that oscillating cycle time period is not prominent. (**m**) Quantification of the A-P to D-V length ratio in S10 and S14 egg chambers with the indicated genetic backgrounds. *n* is the number of samples analysed. Error bars indicate ±s.d. N.S. means no significant difference, while *P*<0.001 means significant difference by student's *t*-test.

To further test the role of cell–matrix adhesion in basal MyoII oscillation, we investigated the genetic interaction between cell–matrix adhesion and Rho1-ROCK signals in control of basal MyoII oscillation. Here a mosaic expression system called FLP-OUT has been used to randomly express different transgenes in follicle cells. Compared with the neighboring WT cells (without mCD8GFP expression), the expression of Rho1 or ROCK active form strongly induces basal MyoII intensity, and either disrupts or enhances MyoII oscillation period, respectively, in mosaic clone cells (with mCD8GFP expression); in the opposite case, the β-integrin or Talin inhibition by RNAi strongly decreases basal MyoII intensity and oscillation period; interestingly, concurrent expression of Rho1 active form and *β-Integrin RNAi*, or ROCK active form and *Talin RNAi*, completely recovers both parameter levels to those observed in WT cells (Fig. 3a–h). Moreover, the inhibition of Rho1 activity by the expression of Rho1 dominant negative form blocks basal MyoII intensity and oscillation, while as previously reported^9^ the enhancement of cell–matrix adhesion by Paxillin expression prominently increases basal MyoII intensity and oscillation period; and concurrent expression of both completely restores both parameter levels to those of WT cells (Fig. 3i–l).

Thus, our findings clearly demonstrated the positive control of Rho1-MyoII activities by cell–matrix adhesion at basal domain of oscillating follicle cells. Consistently, we confirmed the biological role of this signal control in the regulation of tissue elongation^9^. The S10 tissue morphologies affected by the expression of either *β-Integrin RNAi* or *Talin RNAi* can be strongly recovered to those of control when the respective activation of Rho1 or ROCK is introduced back (Fig. 3m). Oppositely, the S10 tissue shape change mediated by Paxillin overexpression is also rescued back to normal shape when Rho1 activity is inhibited (Fig. 3m). In all these cases, the S14 tissue elongation gets partially but significantly recovered (Fig. 3m).

Considering that the inhibition of cell–matrix adhesion has little effect on the rotation speed of egg chambers (Supplementary Fig. 2), our results of basal MyoII oscillation and tissue elongation demonstrated that cell–matrix adhesion strongly governs tissue elongation via the Rho1-MyoII signal cascade.

### Cell–cell adhesion regulates basal Rho1-MyoII distribution

**Figure 4:**
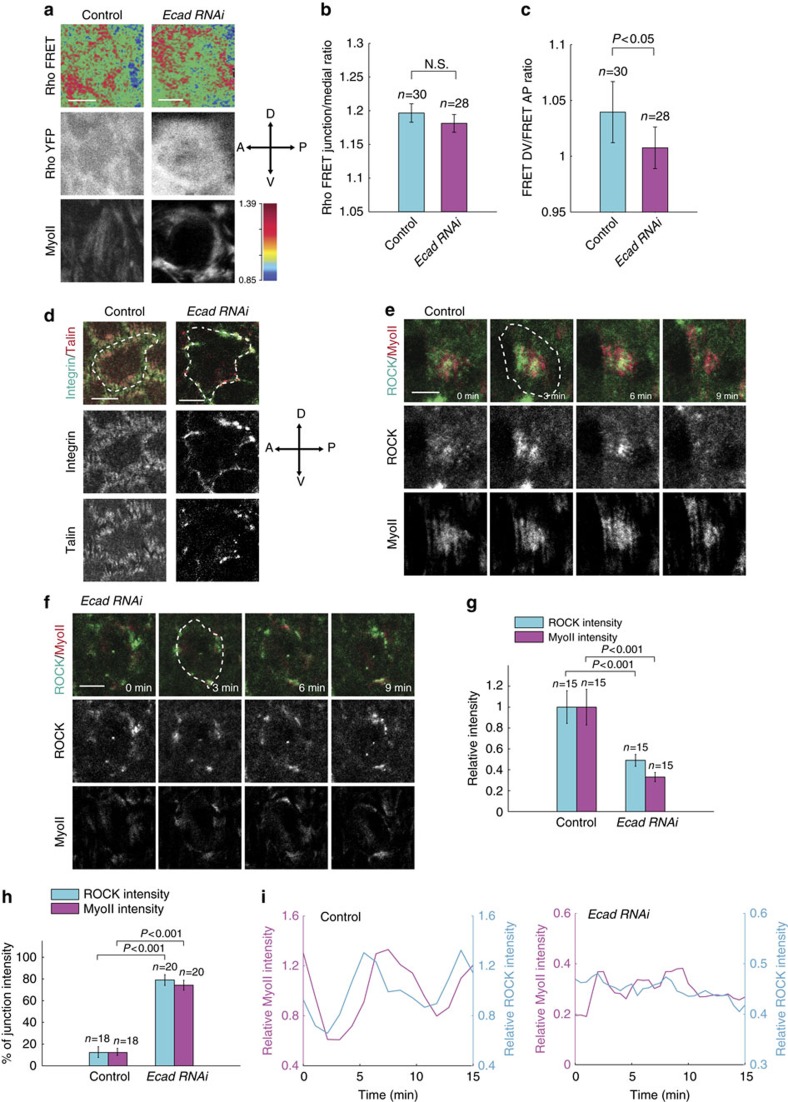
Cell–cell adhesion controls the distribution pattern of basal ROCK and MyoII signals but not the Rho1 activity. (**a**) Representative Rho FRET images, together with YFP channel only and MyoII-mCherry, in the wild type and *E-cadherin RNAi*-expressing follicle cells. Top, processed Rho FRET signal; middle, YFP channel only; bottom, MyoII signal. (**b**) Quantification of the basal junction/medio-basal Rho FRET ratio in these two indicated conditions. (**c**) Quantification of the DV to AP junction Rho FRET ratio in these two indicated conditions. (**d**) Confocal micrographs of β-Integrin-GFP together with Talin-mCherry in follicle cells with the wild type and *E-cadherin RNAi*-expressing genetic backgrounds. Dotted line marks basal junction between follicle cells. (**e**,**f**) Time-lapse series of one representative wild type (**e**) and *E-cadherin RNAi*-expressing (**f**) follicle cell (one cell is marked by dotted line), labelled with ROCK-GFP and MyoII-mCherry. All scale bars are 5 μm. (**g**) Quantification of relative ROCK and MyoII intensities in these two indicated conditions. Average value of ROCK and MyoII intensities in one oscillating cycle has been used as one sample for average quantification. (**h**) Quantification of the relative percentage of ROCK and MyoII intensities distributed at and near basal junction in these two indicated conditions (from the mosaic clones of Supplementary Fig. 6). *n* is the number of samples analysed. All error bars indicate ±s.d. N.S. means no significant difference, while *P*<0.05 and *P*<0.001 mean weak and significant difference by student's *t*-test. (**i**) Quantifications of the dynamic changes of ROCK-GFP and MyoII-mCherry intensities in one oscillating cell with control and *E-cadherin RNAi*-expressing conditions, respectively. In (**i**), the intensities of ROCK and MyoII signals in *E-cadherin RNAi*-expressing (**f**) follicle cells have been normalized to those in control cells (**e**).

We observed that the expression and distribution patterns of E-cadherin are not correlated with MyoII signal at basal domain of follicle cells (Fig. 1c,f), indicating that E-cadherin adhesion might not be involved in the positive control of Rho1 and ROCK signals. Indeed, compared with control cells, the E-cadherin inhibition by RNAi has no significant effect on Rho1 activity at and near basal junction as well as on the β-integrin and Talin intensities at basal domain (Fig. 4a,b and Supplementary Fig. 5a,b,d,e). However, the moderate planar cell polarity of Rho1 activity detected in control cells disappears after the E-cadherin inhibition (Fig. 4c). Consistent with this result, the distributions of β-Integrin and Talin, which are mainly located at the DV axis in control cells, become almost equal at the DV and AP axis, and enriched at basal junction after the E-cadherin inhibition (Fig. 4d and Supplementary Fig. 5a,c,d,f). This data indicates that cell–matrix adhesion redistributes from original focal adhesion sites to near basal junction when basal cell–cell adhesion is inhibited. Consistent with the polarity loss of Rho1 activity and the redistribution of cell–matrix adhesion, we detected the redistribution of ROCK and MyoII signals from medio-basal region to near basal junction after the E-cadherin inhibition (Fig. 4e–h and Supplementary Movie 5). We also observed the reduction of basal ROCK and MyoII intensities and both signal oscillations (Fig. 4e–i). This reduction of both signals, which is somehow inconsistent with the absence of significant change of basal Rho1 activity, could be due to the expansion of both signals from basal domain to more apical regions. When we checked ROCK and MyoII signals at different Z-stack layers, we really detected that both signals range from basal cortical domain to lateral junction in more apical regions after the E-cadherin inhibition; while they are only limited to medio-basal region in the control, *β-Integrin RNAi*-expressing, and *Talin RNAi*-expressing cells (Supplementary Fig. 6a–c). The apical expansion of ROCK and MyoII signals is consistent with the spatial redistribution of Rho1 signal: Rho1 protein and activity are significantly higher at lateral junction in more apical regions after the E-cadherin inhibition, compared with those in control (Supplementary Fig. 6d–f). Taken together, our data indicate that the reduction of basal MyoII oscillation by the E-cadherin inhibition is not due to the change of basal Rho1 activity, but comes from the localization change of ROCK and MyoII signals, and the apical expansion of junctional Rho1 signal.

### Stress fibres and Dia redistribute ROCK and MyoII

**Figure 5:**
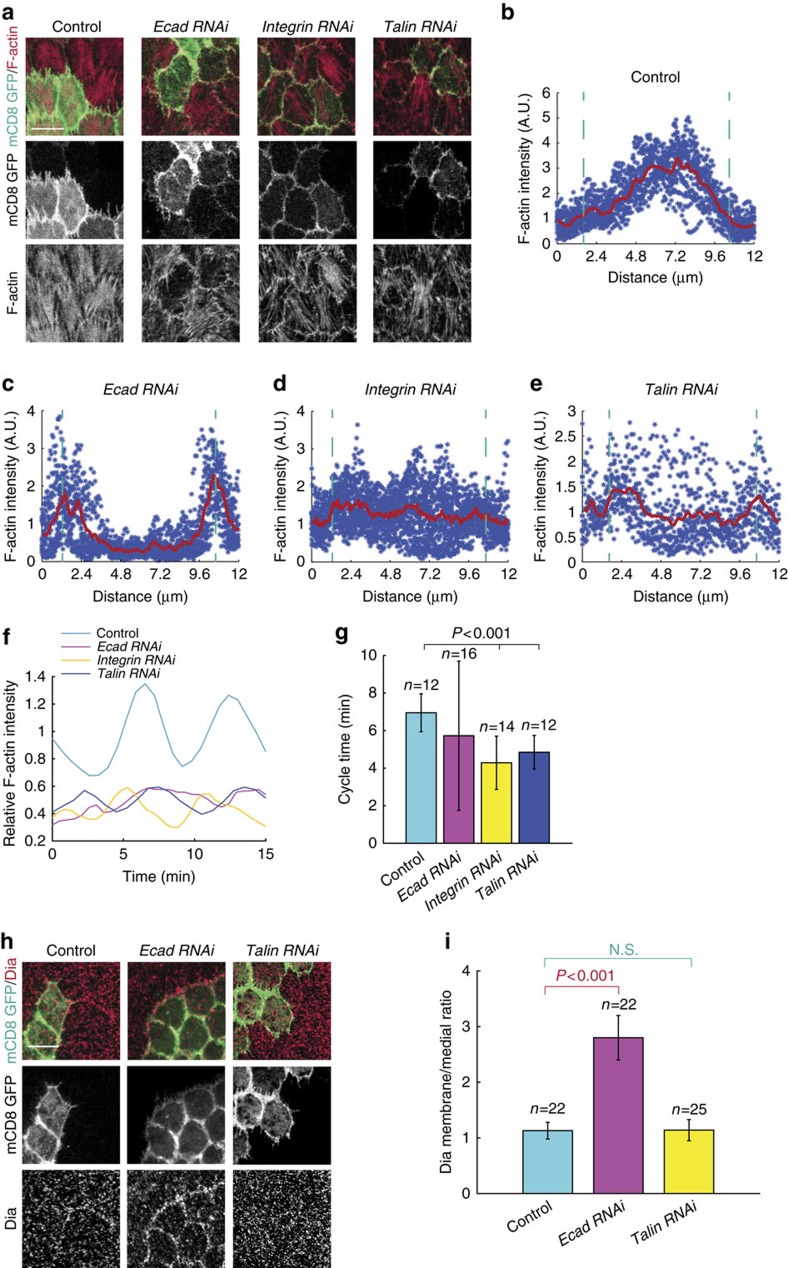
Cell–cell adhesion controls the distribution pattern of basal F-actin and Dia. (**a**) Confocal micrographs of F-actin signal in follicle cell clones expressing the indicated transgenes, marked by coexpression of mCD8GFP. F-actin signals have been assessed by phalloidin staining. (**b**–**e**) Individual mean fluorescent intensities and total averages of basal F-actin signal from linescans across the indicated follicle cells (18<*n*<30) in **a**. Dotted green lines label the anterior and posterior cell junctional membranes. Each blue dot is an individual intensity and red graph is the average value. (**f**) Quantification of the dynamic changes of F-actin intensity in one representative oscillating cell with the indicated genetic backgrounds. F-actin dynamics have been assessed by UtrABD-GFP. Average intensity of F-actin signal in the control oscillating cell is set as 1. (**g**) Quantification of average F-actin oscillating cycle time period in the indicated genetic backgrounds. (**h**) Confocal micrographs of Dia signal in follicle cell clones expressing the indicated transgenes, marked by coexpression of mCD8GFP. Both scale bars are 10 μm. (**i**) Quantification of the membrane to medial Dia intensity ratio in follicle cell clones with the indicated genetic backgrounds. *n* is the number of samples analysed. Error bars indicate ±s.d. N.S. means no significant difference, while *P*<0.001 means significant difference by student's *t*-test.

**Figure 6:**
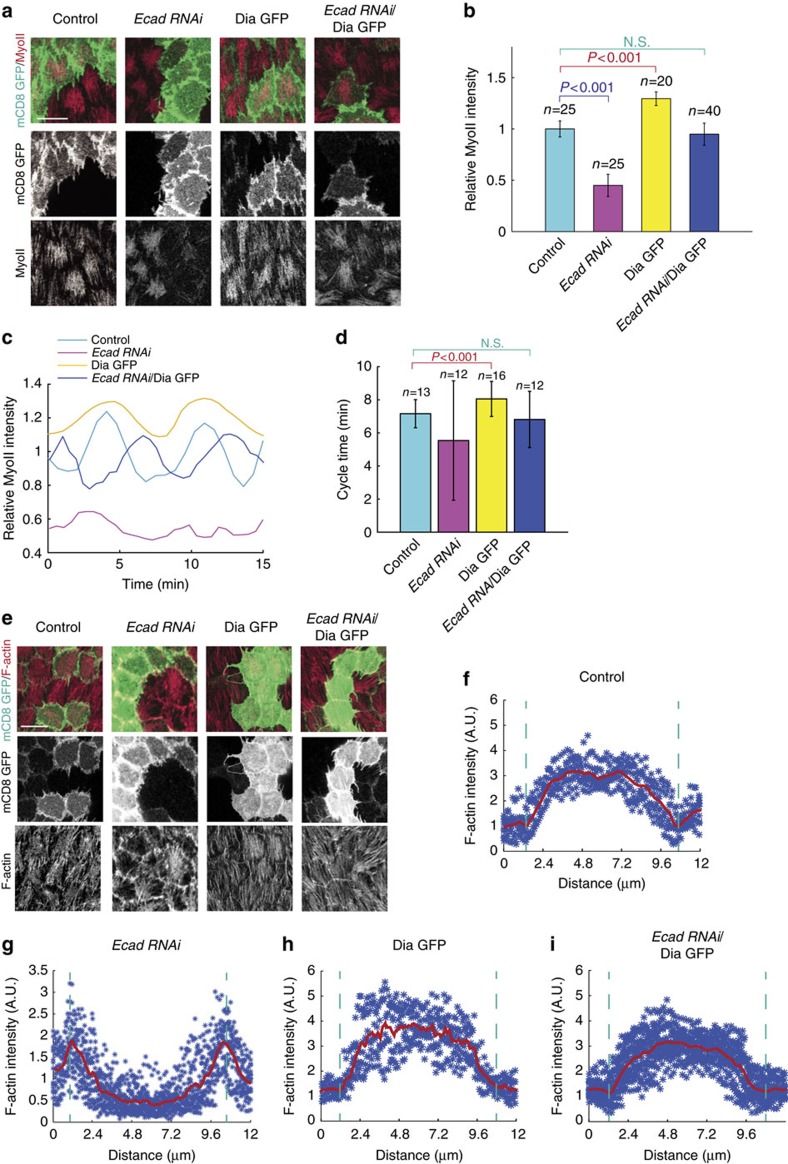
Cell–cell adhesion controls the medio-basal actomyosin network via Dia. (**a**,**e**) Basal views of follicle cell clones expressing the indicated transgenes, marked by coexpression of mCD8GFP. Signals of MyoII (**a**) and F-actin (**e**) have been assessed by MyoII-mCherry and phalloidin staining, respectively. Both scale bars are 10 μm. (**b**) Quantification of relative MyoII intensity in the indicated transgene-expressing GFP-positive cells compared with the GFP-negative wild-type cells in the same sample. (**c**) Quantification of the dynamic changes of MyoII intensity in one representative oscillating cell with the indicated genetic backgrounds. Average intensity of MyoII signal in the control oscillating cell is set as 1. (**d**) Quantification of average MyoII oscillating cycle time period in the indicated genetic backgrounds. *n* is the number of samples analysed. Error bars indicate ±s.d. N.S. means no significant difference, while *P*<0.001 means significant difference by student's *t*-test. (**f**–**i**) Individual mean fluorescent intensities and total averages of basal F-actin signal from linescans across the indicated follicle cells (18<*n*<30) in **e**. Dotted green lines label the anterior and posterior cell junctional membranes. Each blue dot is an individual intensity and red graph is the average value.

The following question is how ROCK and MyoII signals redistribute when basal cell–cell adhesion is inhibited. Here we hypothesized that stress fibres^31^ might have a huge change in their subcellular localization. Thus, we examined the F-actin distribution in the WT condition or after the inhibition of cell–cell or cell–matrix adhesion. In the WT cells, F-actin is mainly located at medio-basal region where MyoII motor is able to load (Fig. 5a,b). When E-cadherin is inhibited, F-actin almost completely disappears from medio-basal region and it is mainly enriched at and near basal junction (Fig. 5a,c), indicating that F-actin might have a dramatic redistribution from medio-basal region to basal junction, rather than our previous finding^9^ that F-actin intensity is simply reduced. However, the inhibition of cell–matrix adhesion by RNAi partially reduces F-actin at medio-basal region and meanwhile weakly increases F-actin at basal junction (Fig. 5a,d,e), compared with the WT and *E-cadherin RNAi*-expressing cells. These data, together with the results of basal Rho1 activity (Figs 2a,b, 4a,b), indicate that cell–matrix and cell–cell adhesions differently control F-actin at medio-basal region: cell–matrix adhesion might control F-actin intensity via its positive control of basal Rho1-ROCK signalling activity, while cell–cell adhesion might control the distribution of F-actin independently of basal Rho1-ROCK signalling activity. Consistent with this hypothesis, we detected that concurrent expression of Rho1 active form and *β-Integrin RNAi*, or ROCK active form and *Talin RNAi*, completely recovers medio-basal F-actin intensity to that observed in WT cells; while concurrent expression of ROCK active form and *E-cadherin RNAi* cannot recover medio-basal F-actin intensity to normal level (Supplementary Fig. 7). This redistribution of stress fibre F-actin after the E-cadherin inhibition is consistent with the localization change of cell-matrix adhesion and the apical expansion of Rho1 (Fig. 4d and Supplementary Fig. 6d–f). A recent study demonstrated that basal F-actin oscillation is similar to that of basal MyoII^32^. Thus, we also determined basal F-actin oscillation in the inhibition of cell–matrix and cell–cell adhesions. We found that the effects on basal F-actin oscillation by these two adhesion inhibitions (Fig. 5f,g) are similar to those on basal MyoII oscillation (Figs 3c,d,g,h, 6c,d). Inhibition of cell–matrix adhesion by either *β-integrin RNAi* or *Talin RNAi* expression strongly decreases the oscillation amplitude and cycle time period of basal F-actin signal, while inhibition of cell–cell adhesion by *E-cadherin RNAi* expression results in the reduced oscillating amplitude and very stochastic cycle time period of basal F-actin and MyoII signals.

A main F-actin regulator in control of stress fibre formation is Dia^33^,^34^. Thus, we next characterized whether Dia might be involved in the distribution change of actin filament. In both control and *Talin RNAi*-expressing cells, Dia levels are slightly higher at and near basal junction than in medio-basal region (Fig. 5h,i). Interestingly, the E-cadherin inhibition by RNAi significantly increases the localization of Dia at basal junction (Fig. 5h,i), indicating that Dia might be the regulator of F-actin subcellular distribution. Thus, we next tested whether Dia overexpression is able to alleviate the *E-cadherin RNAi*-mediated reduction of basal MyoII oscillation via the recovery of F-actin at medio-basal region. Overexpression of WT Dia mildly induces basal MyoII intensity compared with control cells (Fig. 6a,b). However, concurrent expression of WT Dia and *E-cadherin RNAi* strongly recovers basal MyoII intensity to the level observed in control cells (Fig. 6a,b). In addition, the reduced oscillation amplitude and stochastic cycle time period of basal MyoII signal, mediated by the *E-cadherin RNAi* expression, are significantly recovered back to normal levels when WT Dia is concurrently expressed (Fig. 6c,d). Consistent with this recovery, Dia overexpression can also restore the distribution pattern of F-actin from basal junction to medio-basal region in the *E-cadherin RNAi*-expressing cells (Fig. 6e–i).

Taken together, our data demonstrated that Dia functions as the major control of F-actin subcellular distribution and thus basal MyoII oscillation when cell–cell adhesion is inhibited. Redistribution of Dia and F-actin might be related to the reorganization of cell–matrix adhesion from original focal adhesion sites to basal junction, and to the apical expansion of Rho1 activity.

### Adhesion-mediated effects are confirmed by optogenetics

Our observation mainly resulted from the genetic modifications, which are slow to exert their effects and tend to induce side effects. Thus, here we introduced an optogenetic method named as GFP-‘Light-activated reversible inhibition by assembled trap' (abbreviated as GFP-LARIAT)^35^ (Supplementary Fig. 8a) in *Drosophila in vivo* system to photo-inactivate the GFP-tagged adhesion proteins in order to determine the spatiotemporal effect on basal MyoII oscillation. The working mechanism of GFP-LARIAT is to sequester the GFP-target proteins into complexes formed by multimeric proteins and a blue light-mediated heterodimerization module^35^. This sequestration will prevent the GFP-target proteins from their functional region or signal cascade.

The success of LARIAT is highly dependent on the equal amount of CIB-MP and CRY-VHH(GFP) expression levels^35^,^36^. Thus, we used 2A linker system^37^ to drive the expression of both CIB-MP and CRY-VHH(GFP), and we tested a few constructs and identified construct #6 (Supplementary Fig. 8b–d and Supplementary Movies 6–8) that produces the best efficiency in the light-induced GFP-LARIAT. Then, we produced the LARIAT (construct #6 without mCherry), CIB-MP or CRY-VHH(GFP) transgenic flies and tested their respective light-induced effects on MyoII-GFP *in vivo*. Compared with control cells, the expression of CIB-MP has no effect on the MyoII-GFP clustering after light illumination (Supplementary Fig. 9a–c). Although the expression of CRY-VHH(GFP) weakly induces the MyoII-GFP clustering by light, it has no prominent effect on basal MyoII oscillation (Supplementary Fig. 9a–d and Supplementary Movie 9), which is similar to that of control and CIB-MP expression. However, the expression of LARIAT leads to a gradual and significant clustering of MyoII-GFP after the pulsed light illumination; meanwhile, basal MyoII oscillation is completely blocked after MyoII fibres have been aggregated by the light-induced GFP-LARIAT (Supplementary Fig. 9a–d and Supplementary Movie 9). Taken together, this confirmed the feasibility of LARIAT application *in vivo*.

**Figure 7:**
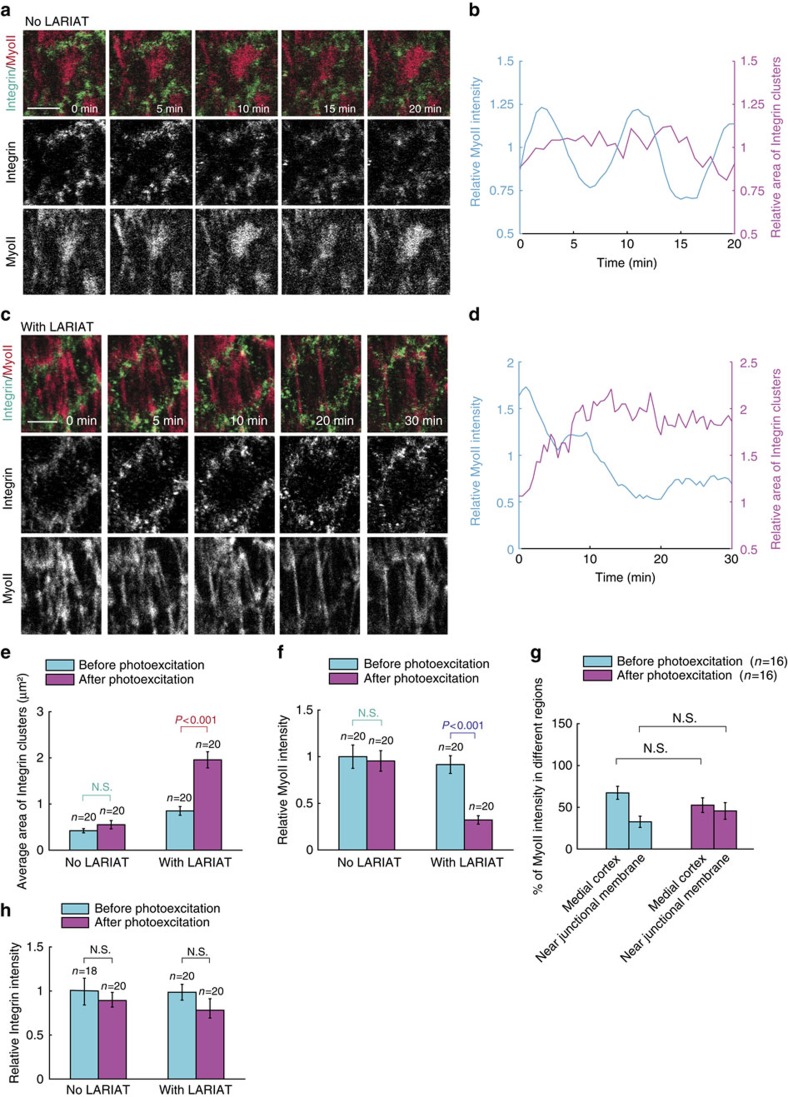
Light-induced β-Integrin-GFP clustering inhibits basal MyoII oscillation. (**a**,**c**) Time-lapse series of one representative wild type (**a**) and LARIAT-expressing (**c**) follicle cell, labelled with β-Integrin-GFP (here is *β-Integrin-GFP*/*β-Integrin-GFP* genotype without no-GFP-tagged wild type *β-Integrin*) and MyoII-mCherry, and illuminated with blue light for 20–30 min at 30 s interval. Both scale bars are 5 μm. (**b**,**d**) Quantifications of the dynamic changes of relative MyoII intensity and relative area of β-Integrin clusters in no LARIAT (**b**) and with LARIAT (**d**) conditions. (**e**,**f**,**h**) Quantifications of average β-Integrin clustering area (**e**) relative MyoII intensity (**f**) and relative β-Integrin intensity (**h**) before and after photoexcitation in the conditions with or without LARIAT expression. (**g**) Percentage of MyoII intensity in different subcellular regions as indicated before and after photoexcitation in the conditions with or without LARIAT expression. *n* is the number of samples analysed. Error bars indicate ±s.d. N.S. means no significant difference, while *P*<0.001 means significant difference by student's *t*-test.

Therefore, we used LARIAT to photo-inactivate cell–matrix or cell–cell adhesions and thus to determine their effects on basal MyoII oscillation. Before the light illumination, β-Integrin and Talin are highly distributed at both sides of MyoII fibres (Fig. 7a and Supplementary Fig. 11a). With the clustering progression, β-Integrin-GFP and Talin-GFP^26^ signals tend to separate from original focal adhesion sites and move inside (Supplementary Fig. 10a–d), indicating that GFP clustering leads to the mislocalization and thus the function change of cell–matrix adhesion proteins. Consistent with this mislocalization, the clustering of β-Integrin-GFP and Talin-GFP gradually and strongly reduces basal MyoII intensity and oscillation compared with no LARIAT-expressing conditions (Fig. 7, Supplementary Fig. 11 and Supplementary Movies 10–13). Thus, our optogenetic results spatiotemporally confirmed the positive control of basal MyoII oscillation by cell–matrix adhesion.

**Figure 8:**
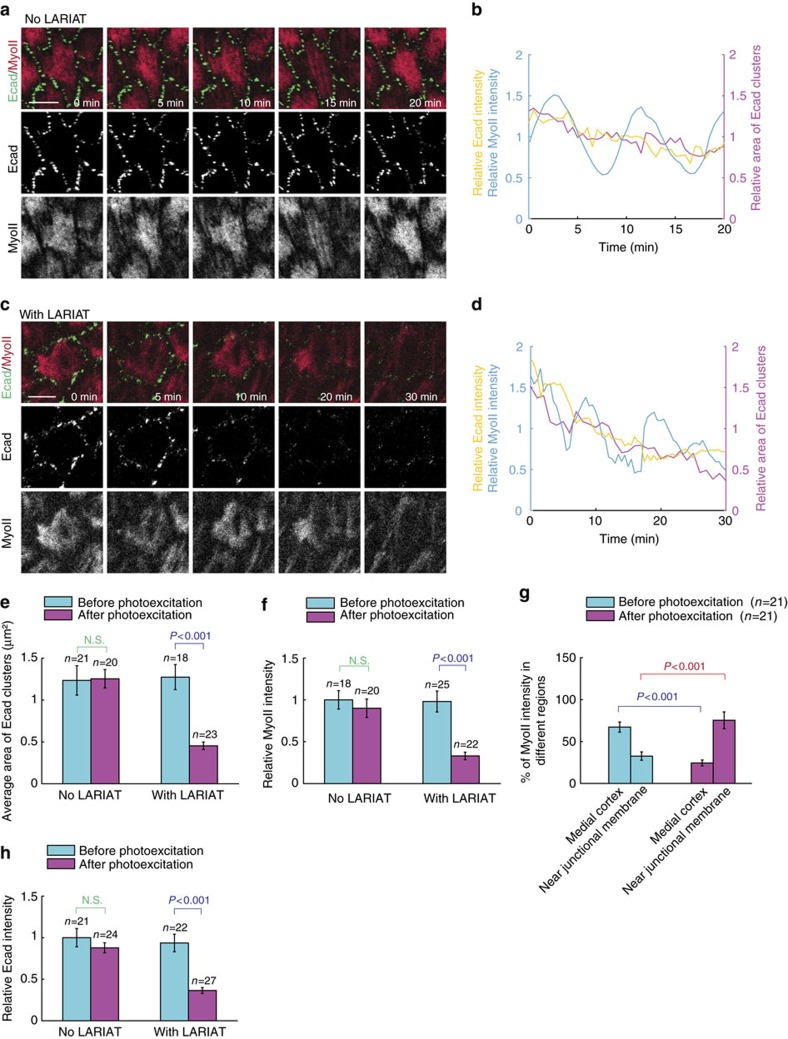
Light-induced E-cadherin-GFP loss results in both reduction and redistribution of basal MyoII oscillation. (**a**,**c**) Time-lapse series of one representative wild type (**a**) and LARIAT-expressing (**c**) follicle cell, labelled with E-cadherin-GFP (here is *E-cadherin-GFP*/*E-cadherin-GFP* genotype without no-GFP-tagged wild-type *E-cadherin*) and MyoII-mCherry, and illuminated with blue light for 20–30 min at 30 s interval. Both scale bars are 5 μm. (**b**,**d**) Quantifications of the dynamic changes of relative E-cadherin/MyoII intensities and relative area of E-cadherin clusters in no LARIAT (**b**) and with LARIAT (**d**) conditions. (**e**,**f**,**h**) Quantifications of average E-cadherin clustering area (**e**) relative MyoII intensity (**f**) and relative E-cadherin intensity (**h**) before and after photoexcitation in the conditions with or without LARIAT expression. (**g**) Percentage of MyoII intensity in different subcellular regions as indicated before and after photoexcitation in the conditions with or without LARIAT expression. *n* is the number of samples analysed. Error bars indicate ±s.d. N.S. means no significant difference, while *P*<0.001 means significant difference by student's *t*-test.

Contrary to the β-Integrin-GFP and Talin-GFP clustering, the E-cadherin-GFP^25^ clustering by LARIAT is not prominent probably because E-cadherin is already distributed as a punctate structure. However, the E-cadherin punctate structure and its mean intensity are gradually and strongly reduced by the light-induced LARIAT compared with no LARIAT-expressing condition (Fig. 8a–e,h and Supplementary Movies 14 and 15). This prominent reduction of E-cadherin levels might come from the clathrin-mediated endocytosis, which has been reported to be highly induced by the antibody-mediated clustering of E-cadherin molecules^38^. With the gradual decrease of E-cadherin at basal junction, basal MyoII oscillation also drops gradually and strongly (Fig. 8a–f and Supplementary Movie 15). Different from the photo-inactivation of cell–matrix adhesion molecules, the loss of basal E-cadherin significantly results in the redistribution of MyoII signal from mainly medio-basal cortex to near basal junction (Figs 7c,g, 8c,g). This redistribution is consistent with the subcellular localization changes of cell–matrix adhesion and basal F-actin: β-Integrin and Talin tend to be equal at the DV and AP axis and also be enriched at basal junction after the significant reduction of basal E-cadherin (Supplementary Fig. 10e–i); and basal F-actin signal is significantly redistributed from medio-basal region to near basal junction after basal E-cadherin-GFP is photo-inhibited (Supplementary Fig. 10j–m). Therefore, our *in vivo* application of LARIAT spatiotemporally confirmed that basal cell–cell adhesion governs the distribution of MyoII and F-actin stress fibres, which is completely different from the effect controlled by cell–matrix adhesion.

## Discussion

Here we demonstrated that the activity and distribution of basal MyoII oscillation are controlled by Integrin-dependent cell–matrix adhesion and E-cadherin-dependent cell–cell adhesion, respectively. The Rho1 activity at and near basal junction and the ROCK/MyoII oscillations at medio-basal cortex are positively regulated by cell–matrix adhesion. Although cell–cell adhesion has no effect on basal Rho1 activity, it strongly regulates the spatial distribution of ROCK and MyoII oscillations at basal domain of follicle cells. Different from our previous observation^9^, cell–cell adhesion seems to exclude F-actin from basal junction and thus limit F-actin mainly at medio-basal region, via the spatial control of F-actin regulator Dia and possibly of Rho1. Thus, cell–matrix adhesion and cell–cell adhesion differentially govern basal MyoII oscillation via the control of either the Rho-ROCK signalling or the distribution of MyoII oscillation (Supplementary Fig. 12).

Consistent with the activity control of basal Rho-ROCK signalling, modified cell–matrix adhesion has a strong effect on tissue elongation. Differently, inhibition of cell–cell adhesion has a very mild effect, whose reason is unclear yet. The redistribution of MyoII oscillation might have some impacts on actomyosin contraction for the underlying tissue elongation. But we cannot exclude the other dramatic effects induced by the loss of E-cadherin adhesion, such as apical-basolateral polarity change. It is completely unknown how other effects have a role in tissue elongation. All these indicate that another but compensatory mechanism might be present in control of tissue shape. In addition, what role does pulsatile actomyosin contractility play? Overexpression of Rho1 active form in follicle cells strongly blocks basal MyoII oscillation, but it enhances basal MyoII intensity and tissue elongation. This effect is similar to that of Ionomyosin treatment, as previously reported^9^. Thus, basal MyoII oscillation might control the gradual and natural tissue shape change, while acute non-pulsatile MyoII contractility achieves the sharp tissue elongation in a much shorter period, which could seriously affect the development of tissue and organ.

It has been well known that cell–matrix adhesion is the major control of the stress fibre formation and the MyoII recruitment in different types of cells growing on extracellular matrix^39^,^40^,^41^. Our results demonstrated that cell–matrix adhesion also governs pulsatile actomyosin network via the Rho1-ROCK signalling pathway. However, several points are still unclear and further studies must elucidate how cell–matrix adhesion regulates junctional Rho1 activity and how this activity is linked with medio-basal ROCK and MyoII oscillations, as well as whether Rho1, cell–matrix adhesion, or adhesion downstream effectors are also pulsatile.

Moreover, our understanding about basal E-cadherin adhesion is very limited. Different from a linear distribution of apical E-cadherin adhesion, basal E-cadherin adhesion presents a punctate distribution in some epithelial cells and endothelial cells^42^,^43^,^44^,^45^. Usually stress fibres are linked with cell–matrix adhesion, however, in endothelial cells or at the free edge of epithelial cells, some types of stress fibre are connected with punctate cadherin adhesion^43^,^45^. In follicle cells, we didnot observe any direct connection between basal E-cadherin adhesion and stress fibres; however, MyoII seems to be excluded from basal junction suggesting that basal E-cadherin adhesion antagonizes stress fibres. This antagonism is somehow similar to the mutual exclusion between actomyosin and apical E-cadherin during cell intercalation^11^,^38^,^46^. The variance of punctate cadherin adhesion might be due to the distribution of E-cadherin adhesion, which is mainly located along stress fibres in endothelial cells and at the free edge of epithelial cells while it is highly enriched along basal junction in follicle cells. Currently, the controlling mechanism and biological role of these punctate cadherin adhesions are largely unknown.

Another commonly important point is the crosstalk between cell–cell and cell–matrix adhesions. This crosstalk includes the level and/or activity control, the shared signalling effectors, and the lateral coupling of both adhesions^47^,^48^,^49^,^50^,^51^,^52^. Here our studies bring novel aspect of this crosstalk. Inhibition of E-cadherin adhesion affects the distribution but not the levels of cell–matrix adhesion, indicating that cell–cell adhesion spatially limits cell–matrix adhesion. Consistent with this, we observed that the inhibition of E-cadherin adhesion results in the redistributions of cell–matrix adhesion and stress fibres, and the apical expansion of Rho1 activity. Here the unclear control of all these changes might be due to the spreading characteristics of cell–matrix adhesion^53^. In individual cells, spreading of cell–matrix adhesion is limited by the balance between the swelling and contraction forces, without the effect from cell–cell adhesion. In collective follicle cells, basal E-cadherin adhesion, together with molecular corset, might spatially limit the spreading of cell–matrix adhesion to restrict the DV polarized actomyosin contraction force within medio-basal region. After the E-cadherin inhibition, this spatial limit is weaken and thus the spreading of cell–matrix adhesion reoccurs, which might strongly break original force balance so that the swelling and contraction forces will be uniformly distributed along basal junction. This hypothesis needs to be confirmed in the future studies.

From the technique view, our studies demonstrated for the first time the successful *in vivo* application of optogenetic LARIAT. Before our success, it was impossible to spatiotemporally control both adhesions *in vivo* by light-inducible or GFP-trap systems^54^,^55^. It is common to modify the actomyosin-mediated biomechanics *in vivo* via laser microsurgery^56^. The success of LARIAT allows to easily and non-invasively study signals and biomechanics *in vivo* with a spatiotemporal resolution. Therefore, it would be interesting and important to extend this optogenetic tool into a broad range of GFP-tagged proteins *in vivo*, which will facilitate our understanding of signals and biomechanics in developmental processes of living organisms.

## Methods

### *Drosophila* stocks and genetics

The following fly stocks were used: Sqh::RLCmyosinII-GFP, Sqh::RLCmyosinII-mCherry^10^ (from Eric E. Wieschaus), Sqh::UtrABD-GFP^11^ (from Thomas Lecuit), DE-cadherin-GFP^25^ (recombination at the locus on the second, from Yang Hong), β-Integrin-GFP^26^ (recombination at the locus on the X, from Nick Brown), Ubi::ROCK-GFP^57^ (from Yohanns Bellaiche), UAS-Paxillin (from Christos G. Zervas), β-Integrin-(WT)-YFP, β-Integrin-(804*stop)-YFP, β-Integrin-(N840A)-YFP, β-Integrin-(N828A)-YFP, β-Integrin-(YY>FF)-YFP, β-Integrin-(G792N)-YFP, and β-Integrin-(L796R)-YFP^27^,^28^ (from Guy Tanentzapf), UAS-*β-Integrin*^*RNAi*^, UAS-*Talin*^*RNAi*^, UAS-*DE-cadherin*^*RNAi*^, UAS-*β-catenin*^*RNAi*^ (from Vienna *Drosophila* RNAi center), Talin-GFP, Talin-mCherry^58^, *ROK[1]*/FM7, UAS-Rho CA, UAS-ROCK CA, UAS-Rho dominant negative, UAS-Dia-GFP, UAS-dsRed (from Bloomington *Drosophila* stock center). *hs*Gal4/CyO, MKRS/TM6B or Sco/Cyo, *hs*Gal4/TM6B flies were used to express UAS lines in follicle cells, in the experiments of Rho FRET and ROCK/MyoII dynamics. For ROCK/MyoII dynamics, Ubi::ROCK-GFP on the third chromosome were combined with *ROK[1]*/FM7 mutant flies in order to prevent the side effect of ectopic ROCK overexpression. Clones were generated using FLP-OUT technique by crossing UAS transgenic flies with (1) P[*hsp*70-flp]; Sqh::RLCmyosinII-mCherry; UAS-mCD8GFP, *Ay*Gal4; (2) P[*hsp*70-flp]; +/+; UAS-mCD8GFP, *Ay*Gal4; (3) P[*hsp*70-flp]; +/+; *Ay*(CD2)Gal4; (4) P[*hsp*70-flp]; UAS-dsRed; *Ay*(CD2)Gal4. To detect ROCK signal in mosaic clones, Ubi::ROCK-GFP on the X chromosome were needed to be combined with UAS transgenic flies firstly, and then clones were generated using FLP-OUT technique by doing the second cross with P[*hsp*70-flp]; +/+; UAS-mCD8RFP, *Ay*Gal4. All stocks and crosses were maintained at room temperature. For signal analysis in mosaic clones, *hs*FLPase was induced for 1 h at 37 °C twice with a 5-h interval, then flies were kept at 18 °C for 2 days and then fattened at 25 °C for overnight before dissection. For the analysis of tissue elongation or follicle rotation, P[*hsp*70-flp]; +/+; *Ay*(CD2)Gal4 or P[*hsp*70-flp]; UAS-dsRed; *Ay*(CD2)Gal4 was used for cross and later heat shock treatment, which can induce >90% clone cells in egg chamber. *hs*FLPase of this mosaic system was induced for 1 h at 37 °C once, then flies were kept at 18 °C for 1–2 days and then fattened at 25 °C for overnight before dissection. For the experiments of Rho FRET and ROCK/MyoII dynamics, *hs*Gal4 flies were incubated at 37 °C for 1 h and flies were kept at 25 °C for 5–6 h before dissection. For the LARIAT experiments, *hs*Gal4 was used to induce the LARIAT expression in ovary follicle cells, and all steps were carried on in dark condition, including cross, maintenance, and heat shock. Flies were treated with heat-shock at 37 °C for 1 h, and then incubated 2–3 h at 25 °C before dissection. *Drosophila* ovaries were dissected and egg chambers were mounted under red light condition before blue light illumination.

### DNA constructs and transgenic fly generation

Full length Rho-FRET complementary DNA^29^ was obtained from Dr Klaus Hahn and amplified by primers as follow: 5′ primer: GGGGACAAGTTTGTACAAAAAAGCAGGCTTCA CCATGGCACACCATCACCACCATC; 3′ primer: GGGGACCACTTTGTACAAGA AAGCTGGGTGTTATCACAAGACAAGGCAACCAG. The PCR product was first cloned into pDONR221 vector (Invitrogen) using BP clonase II (Invitrogen). Then the insertion was recombined into pUASt gateway vector by LR clonase II (Invitrogen). Individual one vector of LARIAT plasmids were generated by In-fusion cloning (Clontech). Briefly, individual pieces of PHR, VHH(GFP), CIB1, MP (CaMKIIα association domain), SNAPtag and dsRedEX2 were PCR amplified starting from PHR-VHH(GFP) and CIB1-mCherry-MP constructs^35^. Each pieces of amplified PCR fragments were cloned into pEGFP-C1 vector or pmCherry-C1 vector (Clontech). A detail primer and cloning information was listed in Supplementary Table 1. The pUASt plasmids of LARIAT with mCherry (construct #8), LARIAT without mCherry (construct #9), CRY2-VHH(GFP) (construct #10), and CIBN-MP (construct #11) were generated by In-fusion cloning (Clontech). Individual pieces were PCR amplified starting from construct #6 vector (pC1-vhhGFP-SNAP-PHR-P2A-CIBN-mCherry-AD). Each pieces of amplified PCR fragments were cloned into pUASt vector. A detail primer and cloning information was listed in Supplementary Table 1.

UAS-Rho FRET and all the UAS-LARIAT series flies were generated by Centro de Biologia Molecular Severo Ochoa (CSIC/UAM) using w1118 fly. To test whether UAS-Rho FRET can phenocopy the role of *Drosophila* Rho1, we did the phenotype rescue experiment of Rho1 LOF mutant flies. *Rho1*^*1B*^/*Rho1*^*1B*^ LOF mutant flies are not viable, while the expression of UAS-Rho FRET driven by *tub*Gal4 can rescue the viability defect from the *Rho1*^*1B*^ LOF mutant (we can get some viable flies with two alleles of *Rho1*^*1B*^, in the condition of UAS-Rho FRET with *tub*Gal4). Flies with UAS-Rho FRET were crossed with *hs*Gal4; sqh::RLCmyosinII-mCherry flies to test the expression pattern and fluorescence level in the indicated conditions (Supplementary Fig. 4). The basal junctional distribution of Rho FRET (Supplementary Fig. 4) is very similar to the basal localization patterns of RhoGEF2-GFP, RhoGEF2 antibody staining, Rho1-GFP and Rho1 antibody staining (Supplementary Fig. 4). Thus, both the rescue result and the similar subcellular distribution patterns indicate that this Rho FRET somehow behaves the same as the wild-type Rho1 in *Drosophila*. Flies with LARIAT (UAS-vhhGFP-SNAP-PHR-P2A-CIBN-AD) were crossed with hsGal4 flies to see the sequestration of GFP-tagged proteins and their effect on mCherry-tagged signals.

### Dissection and mounting of *Drosophila* egg chamber

One- to three-day-old females were fattened on yeast with males for 1–2 days before dissection. *Drosophila* egg chambers were dissected and mounted in live imaging medium (Invitrogen Schneider's insect medium with 20% FBS and with a final PH adjusted to 6.9), by using a similar version of the protocol described in ref. 59. Different from normal mounting condition, the egg chambers were slightly compressed to overcome the endogenous curvature. In this condition, basal oscillation pattern, intensity and period were similar to those in the condition without compression.

### Imaging and photomanipulation

Time-lapse imaging was performed with a Zeiss LSM710 or Leica SP8 confocal microscope with a 40 × , numerical aperture 1.3 inverted oil lens, with a 488-nm argon laser and a 568-nm green HeNe laser. The basal focal plane, which is about 1 μm beneath the basal surface, was selected during live imaging to maximize the basal MyoII intensity. For the dynamics of ROCK-GFP or UtrABD-GFP, the similar basal focal plane was selected to maximize the basal ROCK or F-actin intensity, as basal MyoII dynamic imaging did. The same microscope set-up was used when comparing intensity between different samples. To view signals at different focal planes, images were taken at different Z-stack layers from the basal surface to the apical side. For the dynamics of follicle cell rotational movement, the focal plane, which is centered at follicle cell nuclei, was selected to maximize the nuclear dsRed to better view the localization of individual follicle cells.

FRET images of live cultured egg chambers were acquired with a Zeiss LSM710 microscope, by using a similar version of the protocol described in ref. 60. A 458 nm laser was used to excite the sample. CFP and YFP emission signals were collected through channel I (470–510 nm) and channel II (525–600 nm), respectively. To capture single, high-resolution, stationary images, a 40X/1.3 oil inverted objective was used. CFP and YFP images were acquired simultaneously for most of the experiments. Sequential acquisition of CFP and YFP channels in alternative orders were tested and gave the same result as simultaneous acquisition.

For photoexcitation experiment, live-cell imaging was performed using a Zeiss LSM710 confocal microscope with a 40 × , numerical aperture 1.3 inverted oil lens, with a 488-nm argon laser and a 568-nm green HeNe laser. LARIAT clustering system was effectively induced by the blue light wavelength (400–510 nm) and thus here 488-nm argon laser was used to do the photoexcitation. To avoid the strong photo-bleaching effect on both GFP and RFP signals during photoexcitation, the 488 nm laser was set at 6% power level to do both GFP signal scanning and pulsed photoexcitation of LARIAT optogenetic system in a time-lapse imaging acquisition, taken every 30 s.

### Transfection and image acquisition of cultured cells

HeLa cells were maintained in Dulbecco's modified Eagle's medium (PAA Laboratories GmbH) supplemented with 10% FBS (Invitrogen), 100 U ml^−1^ penicillin and streptomycin at 37 °C and 10% CO_2_. HeLa cells were seeded onto 96-well plate (Corning) for 24 h before transfection. Cells were transfected using jetPRIME reagent (Polyplus) according to the manufacturer's instructions. A total of 200 ng DNA was used in each well of a 96-well plate. Transfection media was replaced after 4 h with fresh media and plate was incubated for 24 h. Images were acquired on an ImageXpress Micro XLS automated epifluorescence microscope (Molecular Devices) using a 20X Plan Fluor objective and a 4.66 megapixel complementary metal-oxide-semiconductor (CMOS) camera with a 16-bit readout. Image analysis was performed using MetaXpress software (Molecular Devices).

### Drug treatments

Egg chambers were dissected in live imaging medium, and then incubated with collagenase (1,000 Units ml^−1^ CLSPA; Worthington Biochemical Corp.) dissolved in the final volume of 100 μl culturing medium for 20 min before being mounted for imaging. For the chemical inhibition of Rho1 activity, dissected egg chambers were incubated with two Rho inhibitors: C3 exoenzyme^61^ (Enzo Life Sciences) at 10 μg μl^−1^ or Rhosin^62^ (Merk) at 250 μM for 20 min before being mounted for imaging.

### Image processing and data analysis

Images were processed with MATLAB and Image J. For all images the background (intensity of area without sample) was subtracted.

Image J were used to calculate the intensity of an individual cell as the average value of all pixels within the cell area. In the time-lapse experiments, images were processed by MATLAB to correct photo-bleaching automatically. For dual-colour imaging, the intensities were calculated from manually outlined cell areas if membrane-fluorescent protein was not present to mark cell boundaries.

For the Rho FRET image, CFP and YFP images were first processed by ImageJ software. A background region of interest was subtracted from the original image. The YFP images were registered to CFP images using the TurboReg plugin. Gaussian smooth filter was then applied to both channels. The YFP image was thresholded and converted to a binary mask with background set to zero. The final ratio image was generated with the MATLAB program, during which only the unmasked pixel was calculated^60^. To determine the FRET signal ratio between basal junction and medio-basal region, each follicle cell was separated into junctional region and medial region (based on the MyoII distribution region in control cells), and then the FRET signals in either region were analysed using MATLAB.

For the quantification of egg chamber rotation, the rotation speed of follicle cells was measured from the time-lapse images of S6-S7 egg chambers expressing nuclear dsRed driven by FLP-OUT system. The time-lapse positions of individual follicle cell nuclear center have been automatically tracked by MATLAB and the migration speed has been automatically calculated also by MATLAB. Migration speed (each dot in Supplementary Fig. 2) was obtained from the average of each follicle cell nuclear movement in the same egg chamber.

For the phalloidin image analysis, individual mean fluorescent intensity of F-actin was from around a 10 pixels—linescan across the indicated follicle cells from anterior to posterior axis. Each dot is an individual mean intensity along this linescan and the average intensity was calculated from all follicle cells in the same genotype for this quantification. All were calculated automatically by MATLAB.

The distribution of oscillation periods was generated by measuring the intervals between each pair of adjacent peaks. We applied autocorrelation to calculate the period of a time series with different time offsets. This method averages out irregularities in the sequence and gives a similar average period. We found that autocorrelation was more robust and provided better results in analysing irregular signals with a small amplitude, in some genetic backgrounds with strong reduction or enhancement of MyoII intensity compared with control^9^: genetic backgrounds of *β-Integrin RNAi*, *Talin RNAi*, ROCK active form, *ROCK RNAi*, WT Dia overexpression, and Paxillin overexpression can give the similar periods; genetic background of *E-cadherin RNAi* gives very stochastic periods from very short to long time window; however, this method of autocorrelation cannot give any detectable oscillating period for genetic backgrounds of Rho1 active form or Rho1 dominant negative. To quantify the average oscillating time period, the 25 to 30 minute-dynamic intensity of the *n* individual cells (*n* is indicated in Fig. 3d,h,i, Fig. 5g, Fig. 6d) from four independent LS9 egg chambers was tracked, then average oscillating cycle time of each individual cell was calculated by autocorrelation method. Finally, the oscillating cycle time for these n individual cells in each genetic background was represented as mean±s.d.

The signal intensity ratio of the DV/AP polarity was calculated by the quantification of total intensity distributed at the DV axis relative to that at the AP axis. Tissue elongation was measured by the AP to DV length ratio of S10 and S14 egg chambers.

For the quantification of cluster formation in mammalian cells, clusters were defined as discrete puncta of fluorescence with criteria of fluorescence intensity (2,500–4,095 arbitrary units), size (>0.2 μm^2^) and circularity (0.5–1.0 arbitrary units). The area of clusters per cells was measured with METAMORPH^35^. For the quantification of cluster formation during the photoexcitation of LARIAT system in *Drosophila* follicle cells, all time series of GFP images were automatically adjusted by MATLAB to the initial image intensity in order to reduce the effect of photo-bleaching. Then background noise was subtracted from the adjusted images before the measurement of clusters. Clusters were defined as discrete puncta of fluorescence with criteria of fluorescence intensity (1,200–4,095 arbitrary units), size (>0.2 μm^2^) and circularity (0.35–1.0 arbitrary units). The area of clusters per cells was measured with MATLAB.

### Immunohistochemistry

*Drosophila* ovaries were dissected in Schneider's medium and fixed with 4% paraformaldehyde in PBS (phosphate-buffered saline) for 20 min. After fixation, the egg chambers were rinsed with PBST (PBS with 0.3% Triton X-100) three times. The egg chambers were incubated with various first antibodies normally overnight in cold room. Anti-β-Integrin antibody (mouse CF.6G11, 1:50 dilution), anti-Rho1 antibody (p1D9, 1:50 dilution), and anti-Armadillo antibody (mouse N27A1, 1:50 dilution) were from the Developmental Studies Hybridoma Bank. Anti-Dia antibody (1:5,000) was a gift from Steve Wasserman. Anti-RhoGEF2 antibody (1:1,000) was a gift from Jörg Großhans. Secondary antibodies conjugated with Alex-561 and Alexa-647 (Molecular Probes) were used in 1:400 dilutions. Alexa-561-conjugated phalloidin (1:300 dilution; Invitrogen) was used for F-actin staining. Samples were imaged on a Zeiss LSM710 or Leica SP8 confocal microscope.

### Statistics

All data have been presented as mean±s.d. Statistical analysis to compare results among groups was carried out by Student's *t*-test with two distribution tails. A value of *P*<0.05 was considered to be statistically significant while a value of *P*<0.001 was considered to be remarkable statistically significant.

### Code availability

The codes used for analyses of different images (including FRET and LARIAT) are available from the corresponding author on reasonable request.

### Data availability

The data sets generated during and/or analysed during the current study are available from the corresponding author on reasonable request.

## Additional information

**How to cite this article:** Qin, X. *et al*. Cell-matrix adhesion and cell-cell adhesion differentially control basal myosin oscillation and *Drosophila* egg chamber elongation. *Nat. Commun.* **8,** 14708 doi: 10.1038/ncomms14708 (2017).

**Publisher's note:** Springer Nature remains neutral with regard to jurisdictional claims in published maps and institutional affiliations.

## Supplementary Material

Supplementary Information Supplementary Figures, Supplementary Table and Supplementary Reference

Supplementary Movie 1 Time-lapse series of the representative wild type follicle cell, labelled with ROCKGFP and MyoII-mCherry. Time interval is 1 min, and scale bar is 5 μm.

Supplementary Movie 2 Time-lapse series of the representative β-Integrin RNAi-expressing follicle cell, labelled with ROCK-GFP and MyoII-mCherry. Time interval is 30 sec, and scale bar is 5 μm

Supplementary Movie 3 Time-lapse series of the representative Talin RNAi-expressing follicle cell, labelled with ROCK-GFP and MyoII-mCherry. Time interval is 30 sec, and scale bar is 5 μm.

Supplementary Movie 4 Time-lapse series of the representative Paxillin-expressing follicle cell, labelled with ROCK-GFP and MyoII-mCherry. Time interval is 30 sec, and scale bar is 5 μm.

Supplementary Movie 5 Time-lapse series of the representative E-cadherin RNAi-expressing follicle cell, labelled with ROCK-GFP and MyoII-mCherry. Time interval is 30 sec, and scale bar is 5 μm.

Supplementary Movie 6 Time-lapse series of the HeLa cells transfected with green GFP and two-vector construct (control to show positive effect) and illuminated with blue light. Time interval is 10 sec.

Supplementary Movie 7 Time-lapse series of the HeLa cells transfected with green GFP and #1 one-vector construct (negative effect) and illuminated with blue light. Time interval is 10 sec.

Supplementary Movie 8 Time-lapse series of the HeLa cells transfected with green GFP and #6 one-vector construct (maximal positive effect) and illuminated with blue light. Time interval is 10 sec.

Supplementary Movie 9 Time-lapse series of the representative MyoII-GFP signals in the wild type, CIB1-MP-expressing, CRY2-VHH(GFP)-expressing, and CIB1-MP and CRY2-VHH(GFP)-coexpressing (LARIAT) follicle cells, and illuminated with blue light. Time interval is 30 sec, and scale bar is 5 μm.

Supplementary Movie 10 Time-lapse series of the representative wild type follicle cell, labelled with β-Integrin-GFP and MyoII-mCherry, and illuminated with blue light. Time interval is 30 sec, and scale bar is 5 μm.

Supplementary Movie 11 Time-lapse series of the representative LARIAT-expressing follicle cell, labelled with β-Integrin-GFP and MyoII-mCherry, and illuminated with blue light. Time interval is 30 sec, and scale bar is 5 μm.

Supplementary Movie 12 Time-lapse series of the representative wild type follicle cell, labelled with TalinGFP and MyoII-mCherry, and illuminated with blue light. Time interval is 30 sec, and scale bar is 5 μm.

Supplementary Movie 13 Time-lapse series of the representative LARIAT-expressing follicle cell, labelled withTalin-GFP and MyoII-mCherry, and illuminated with blue light. Time interval is 30 sec, and scale bar is 5 μm.

Supplementary Movie 14 Time-lapse series of the representative wild type follicle cell, labelled with Ecadherin-GFP and MyoII-mCherry, and illuminated with blue light. Time interval is 30 sec, and scale bar is 5 μm.

Supplementary Movie 15 Time-lapse series of the representative LARIAT-expressing follicle cell, labelled with E-cadherin-GFP and MyoII-mCherry, and illuminated with blue light. Time interval is 30 sec, and scale bar is 5 μm.
