# Supplementary material for: Cell-matrix adhesion and cell-cell adhesion differentially control basal myosin oscillation and Drosophila egg chamber elongation
Source: Nat Commun. 2017 Apr 13;8:14708. doi: 10.1038/ncomms14708 (PMC5399299; doi:10.1038/ncomms14708)
Supplement: Supplementary Information — Supplementary Figures, Supplementary Table and Supplementary Reference [file ncomms14708-s1.pdf]

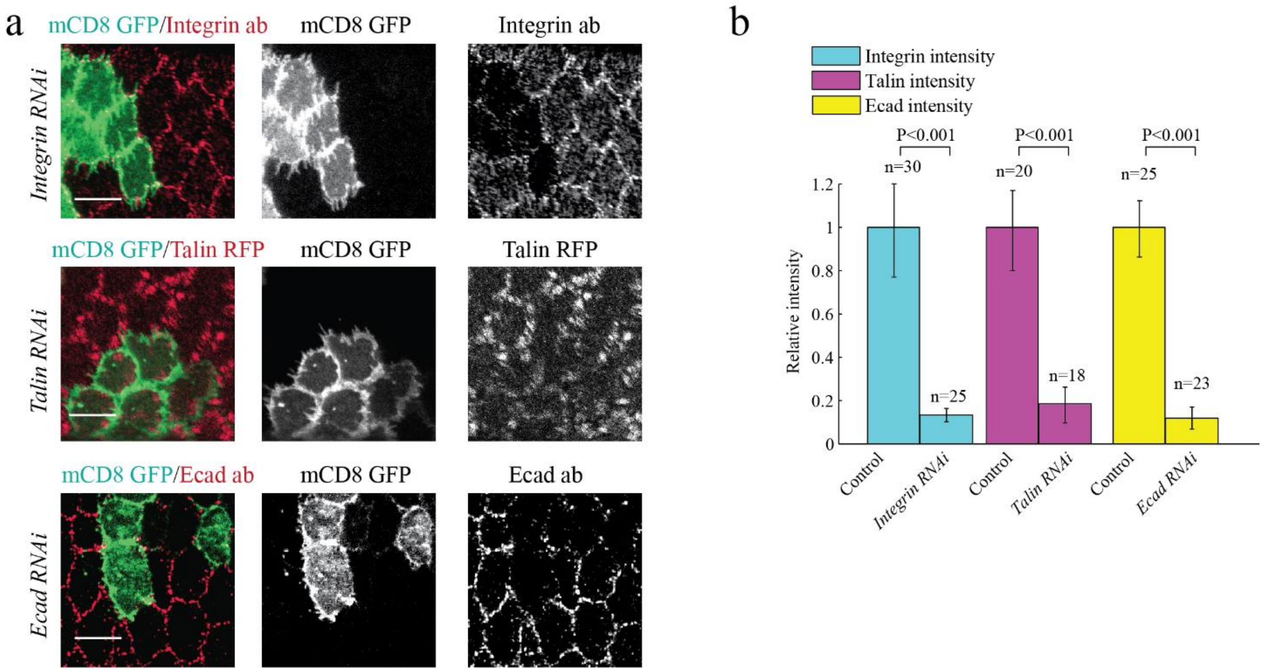

Supplementary Figure 1. Confirmation of knockdown efficiency.

a. Confocal micrographs of the indicated signals in follicle cell clones expressing *β-Integrin RNAi*, *Talin RNAi* and *E-cadherin RNAi*, respectively, marked by coexpression of mCD8GFP. All scale bars are 10 μm. **b.** Relative intensity quantification of the indicated signals in *β-Integrin RNAi*-expressing, *Talin RNAi*-expressing and *E-cadherin RNAi*-expressing follicle cells, compared with control cells. n is the number of samples analyzed. Error bars indicate ±s.d. P<0.001 means significant difference by student's *t*-test.

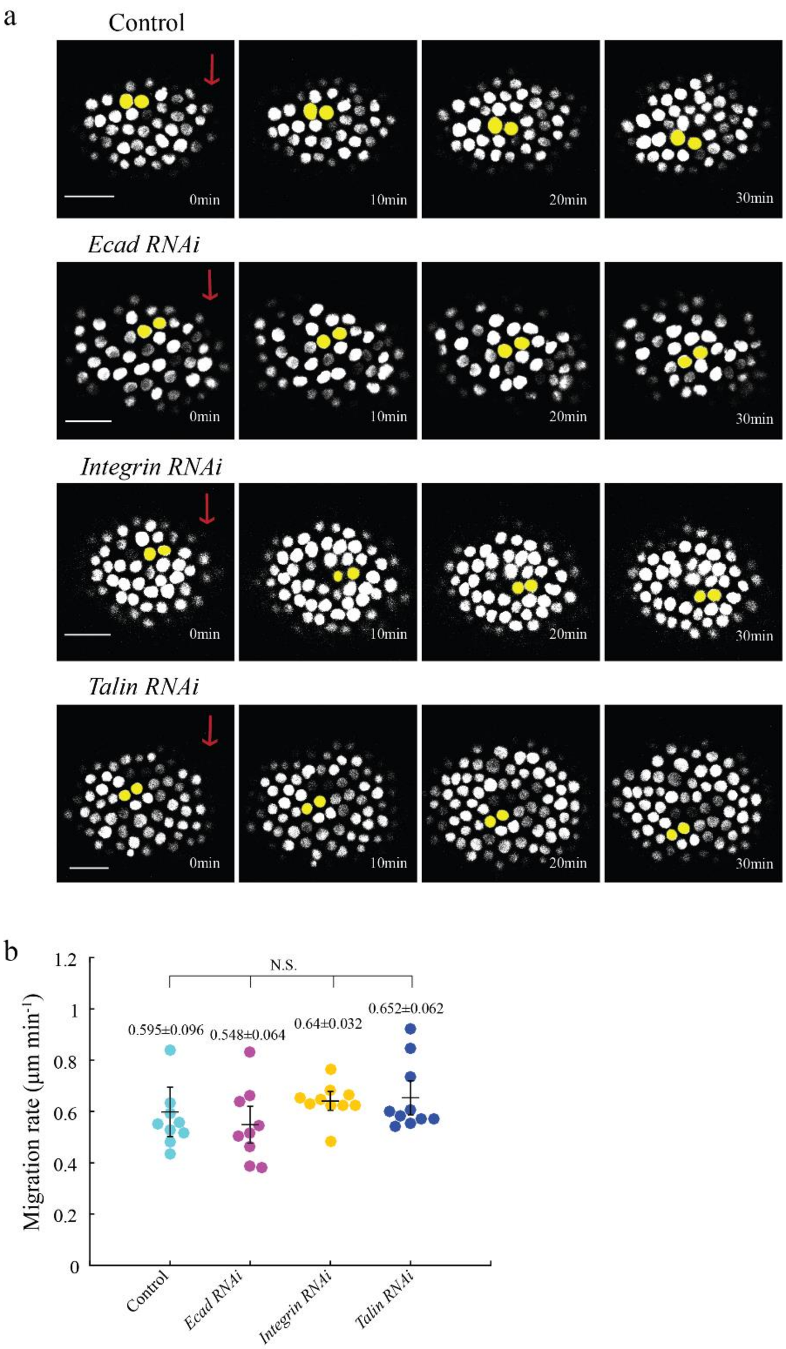

Supplementary Figure 2. Inhibitions of cell-matrix adhesion and cell-cell adhesion have the limited effects on follicle cell rotation.

a. Time-lapse series of one representative egg chamber rotation in the wild type, *β-Integrin RNAi*-expressing, *Talin RNAi*-expressing and *E-cadherin RNAi*-expressing genetic backgrounds, labelled with nuclear dsRed. Two cells in each egg chamber have been marked as yellow color to view tissue rotation. Red arrow indicates the rotation direction. All scale bars are 20 μm. **b.** Quantification of the follicle cell migration speed of individual S6-7 egg chambers in the indicated genetic backgrounds. Individual data points, mean ± s.d. N.S. means no significant difference by student's *t*-test.

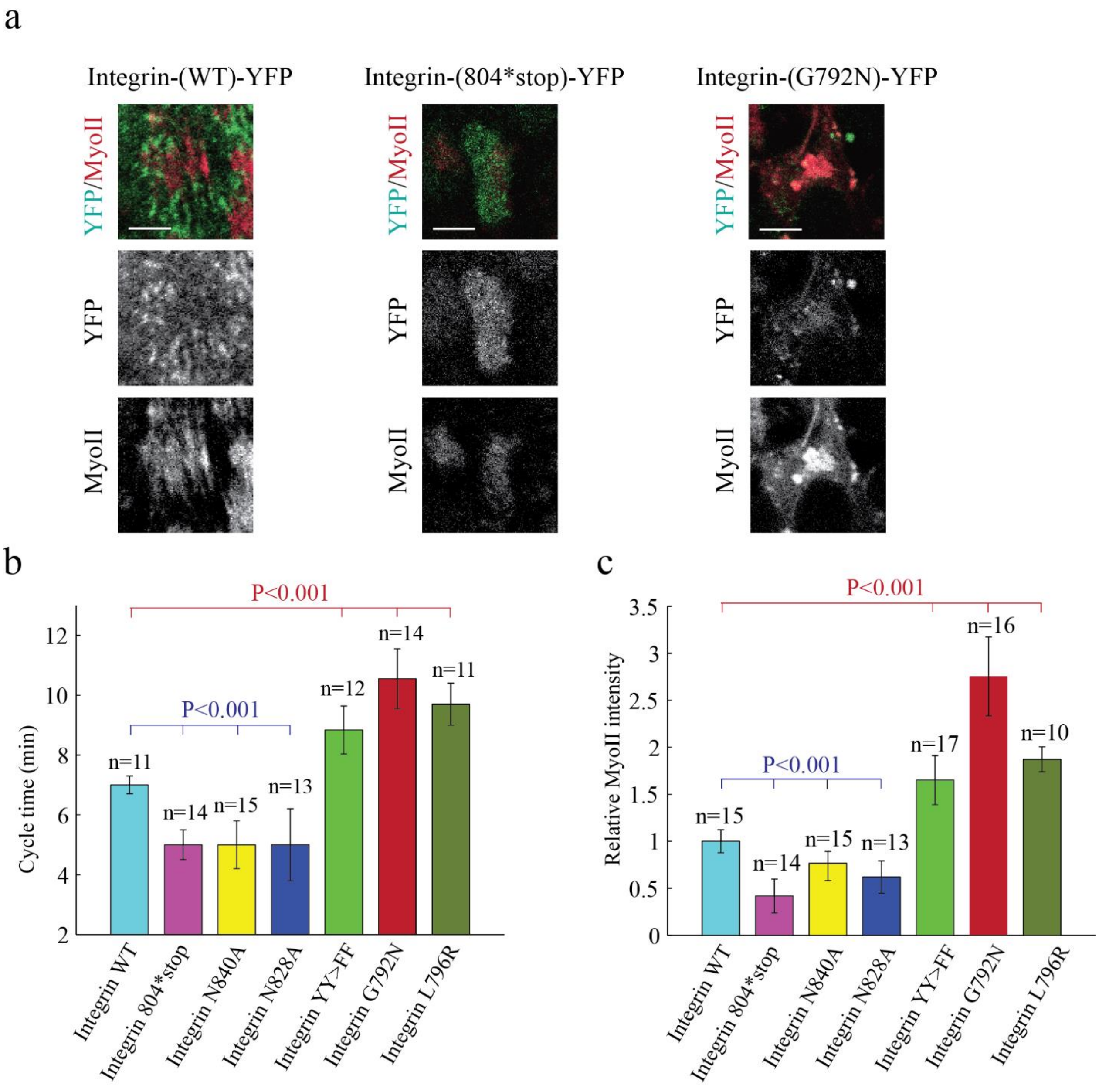

Supplementary Figure 3. Modification of β-Integrin activity affects basal MyoII oscillation.

a. Basal view of follicle cells expressing the indicated β-Integrin-YFP transgenes. All scale bars are 5 μm. **b.** Quantification of the average time period (minute) of basal MyoII oscillation cycle in follicle cells with the expression of indicated β-Integrin-YFP transgenes. **c.** Quantification of relative MyoII intensity in the indicated genetic backgrounds. n is the number of samples analyzed. Error bars indicate ±s.d. P<0.001 means significant difference by student's *t*-test.

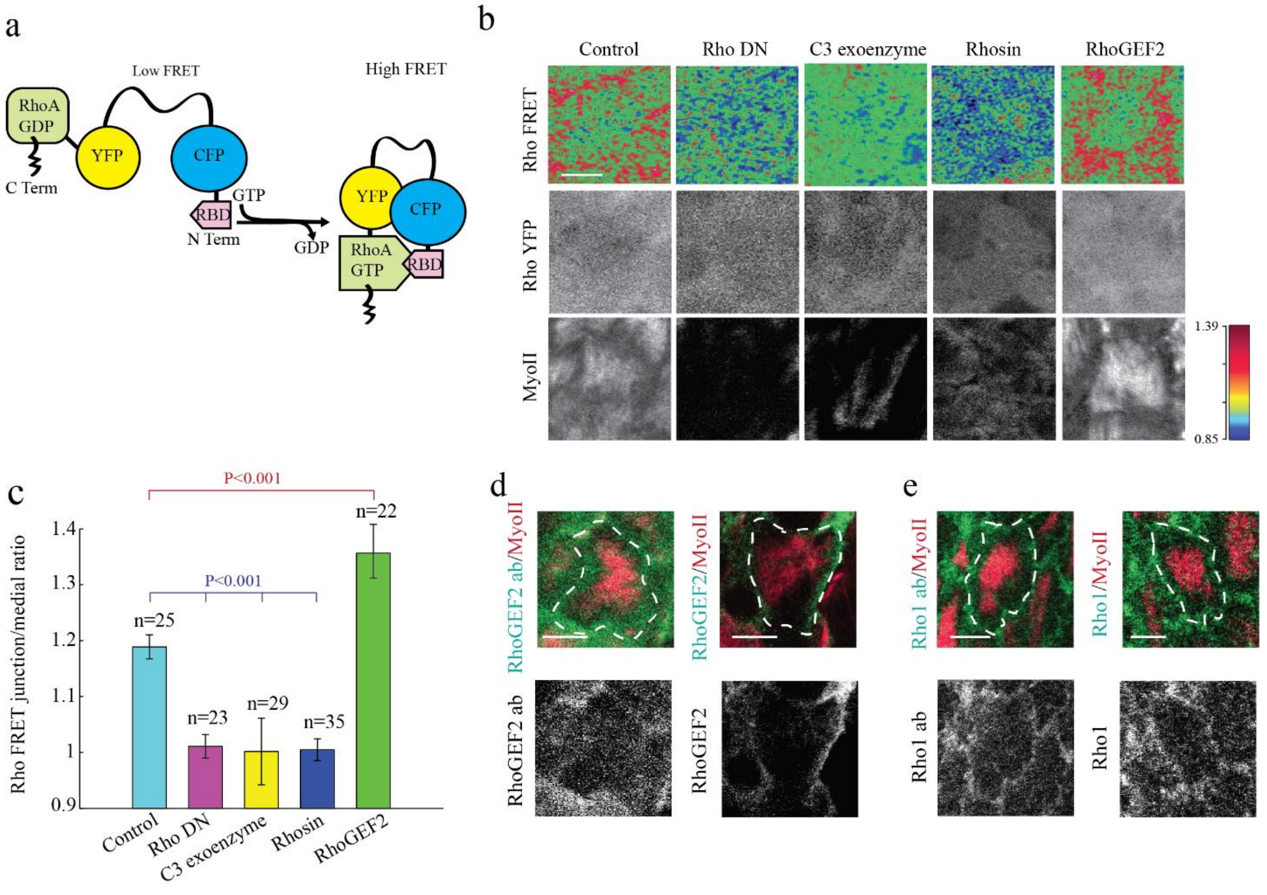

Supplementary Figure 4. Feasibility confirmation of the Rho FRET biosensor.

a. Schematic representation of the Rho FRET probe before and after activation. **b.** Representative Rho FRET images, together with YFP channel only and MyoII-mCherry, in wild type follicle cell, Rho DN-expressing follicle cell, follicle cells with the treatment of C3 exoenzyme and Rhosin (two different Rho inhibitors), and RhoGEF2-expressing follicle cell, respectively. Top, processed Rho FRET signal; middle, YFP channel only; bottom, MyoII signal. **c.** Quantification of the Rho FRET ratio between basal junction and medio-basal region in all indicated conditions. n is the number of samples analyzed. Error bars indicate ±s.d. $P<0.001$ means significant difference by student's *t*-test. The basal junctional distribution of Rho FRET activity is similar to the basal junctional localization patterns of RhoGEF2 (**d**) and Rho1 (**e**) signals, compared with MyoII signal distributed mainly at medio-basal region. **d, e.** Basal views of MyoII signal together with RhoGEF2 signal (**d**) and Rho1 signal (**e**) (one cell is marked by dotted line) detected by antibody staining and fluorescent protein as indicated, respectively. All scale bars are 5 μm.

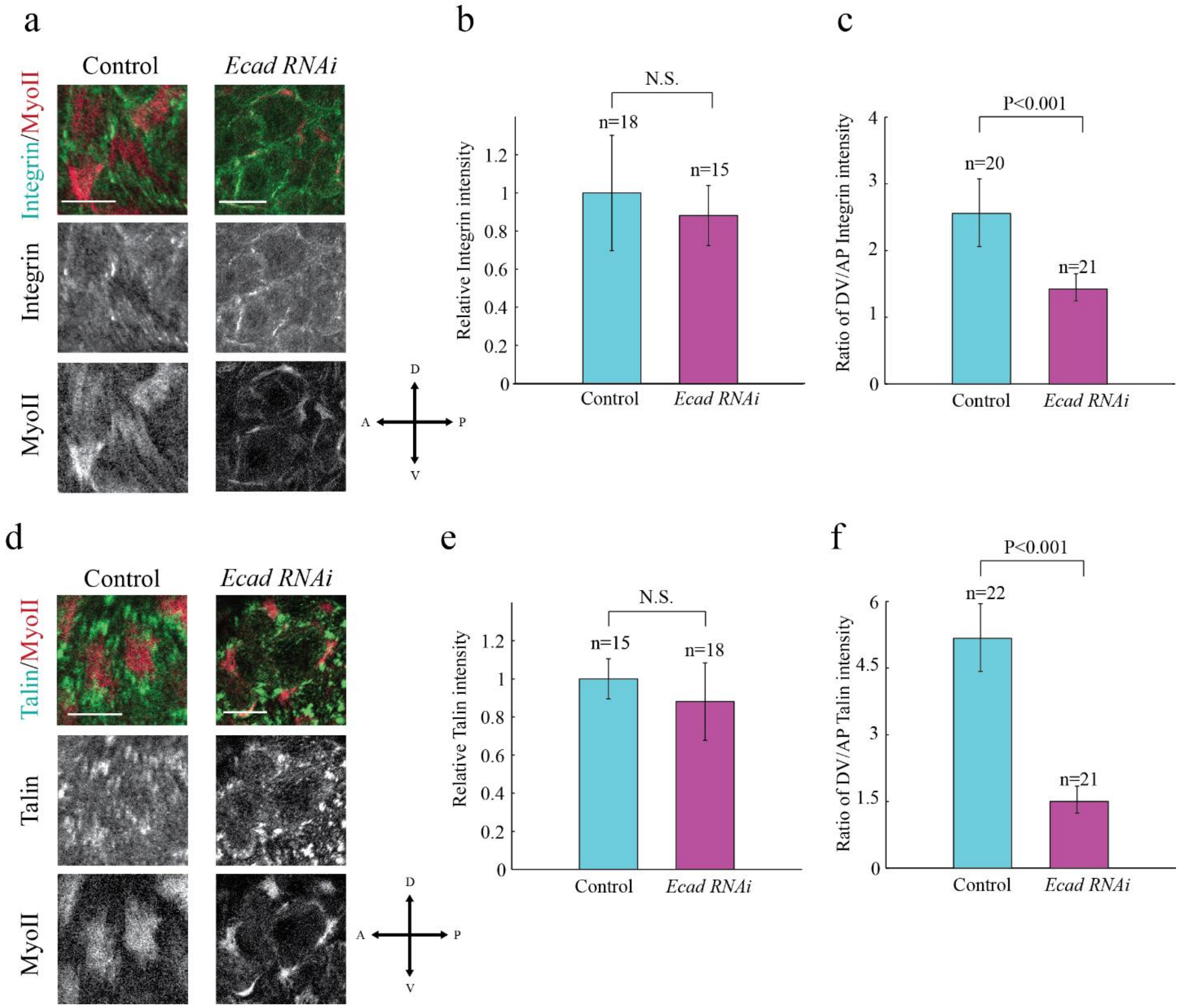

Supplementary Figure 5. Inhibition of cell-cell adhesion changes the distribution but not the intensities of cell-matrix adhesion.

a, d. Confocal micrographs of β-Integrin-GFP (**a**) and Talin-GFP (**d**) together with MyoII-mCherry in follicle cells with the wild type and *E-cadherin RNAi*-expressing genetic backgrounds. All scale bars are 10 μm. **b, e.** Quantifications of relative β-Integrin (**b**) and Talin (**e**) intensities in the indicated genetic backgrounds. **c, f.** Ratio of the DV/AP β-Integrin (**c**) and Talin (**f**) intensities in the indicated genetic backgrounds. n is the number of samples analyzed. Error bars indicate ±s.d. N.S. means no significant difference, while P<0.001 means significant difference by student's *t*-test.

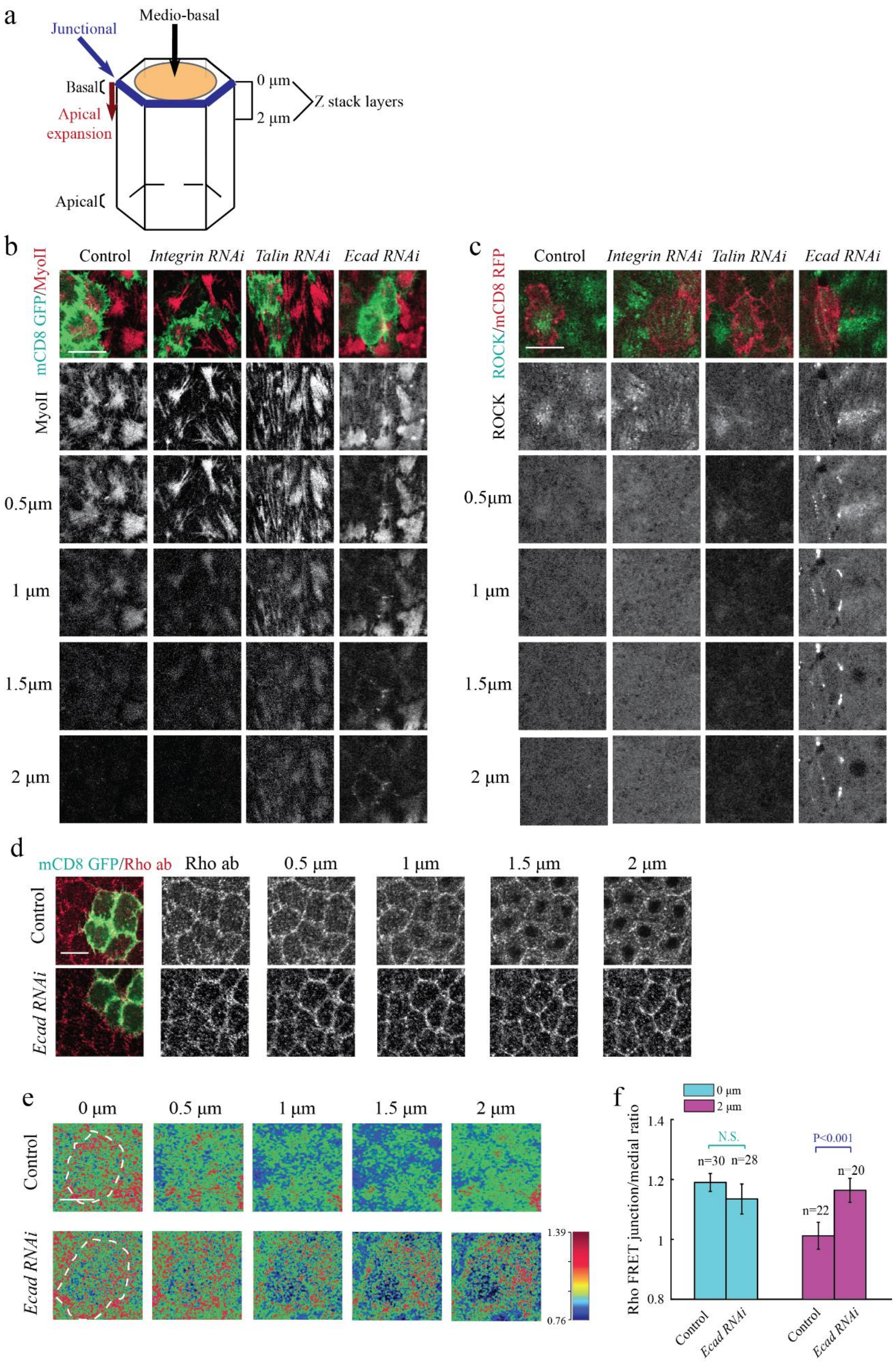

a
Medio-basal
Junctional
Basal
Apical expansion
0 µm
2 µm
Z stack layers
Apical
b
Control
Integrin RNAi
Talin RNAi
Ecad RNAi
mCD8 GFP/MyoII
MyoII
0.5µm
1 µm
1.5µm
2 µm
c
Control
Integrin RNAi
Talin RNAi
Ecad RNAi
ROCK/mCD8 RFP
ROCK
0.5µm
1 µm
1.5µm
2 µm
d
mCD8 GFP/Rho ab
Rho ab
0.5 µm
1 µm
1.5 µm
2 µm
Control
Ecad RNAi
e
0 µm
0.5 µm
1 µm
1.5 µm
2 µm
Control
Ecad RNAi
1.39
0.76
f
0 µm
2 µm
Rho FRET junction/medial ratio
1.4
1.2
1
0.8
N.S.
P<0.001
n=30
n=28
n=22
n=20
Control
Ecad RNAi
Control
Ecad RNAi

Supplementary Figure 6. Inhibition of cell-cell adhesion expands the distribution of ROCK, MyoII and Rho1 signals from basal domain to more apical regions.

a. Schematic representation of basal junctional and medio-basal regions of an individual follicle cell, and apical expansion of signals. **b, c, d.** Confocal micrographs of MyoII (**b**), ROCK (**c**) and Rho1 (**d**) signals from basal to more apical layers in follicle cell clones expressing the indicated transgenes, marked by coexpression of mCD8GFP (**b, d**) or mCD8RFP (**c**), respectively. Images are shown at various Z-stack layers from basal (0 μm) to more apical regions (0.5 to 2 μm). All scale bars are 10 μm. **e.** Representative Rho FRET images from basal to more apical layers in the wild type and *E-cadherin RNAi*-expressing follicle cells (one cell is marked by dotted line). Scale bar is 5 μm. **f.** Quantification of the basal junction/medio-basal Rho FRET ratio at 0 μm and 2 μm Z stack layers in these two indicated conditions. n is the number of samples analyzed. Error bars indicate ±s.d. N.S. means no significant difference, while $P<0.001$ means significant difference by student's *t*-test.

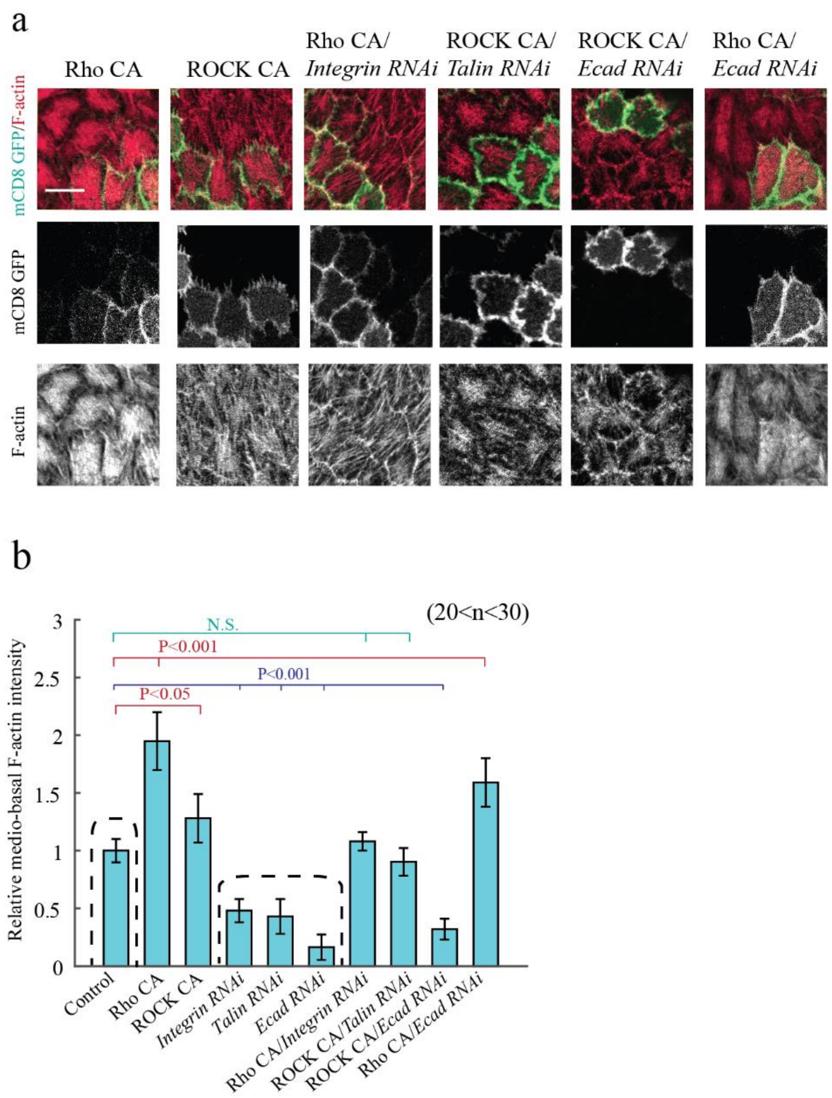

Supplementary Figure 7. Cell-matrix but not cell-cell adhesion controls the medio-basal distribution of F-actin signal via the basal Rho1 and ROCK activities.

a. Confocal micrographs of F-actin signal in follicle cell clones expressing the indicated transgenes, marked by coexpression of mCD8GFP. F-actin signal has been assessed by phalloidin staining. Scale bar is 10 µm. **b.** Quantification of relative medio-basal F-actin intensity in the indicated transgene-expressing GFP-positive cells compared with the GFP-negative wild type cells in the same sample. Representative images of the dashed results are available in Fig.5a. n is the number of samples analyzed. Error bars indicate ±s.d. N.S. means no significant difference, while $P<0.05$ and $P<0.001$ mean weak and significant difference by student's *t*-test.

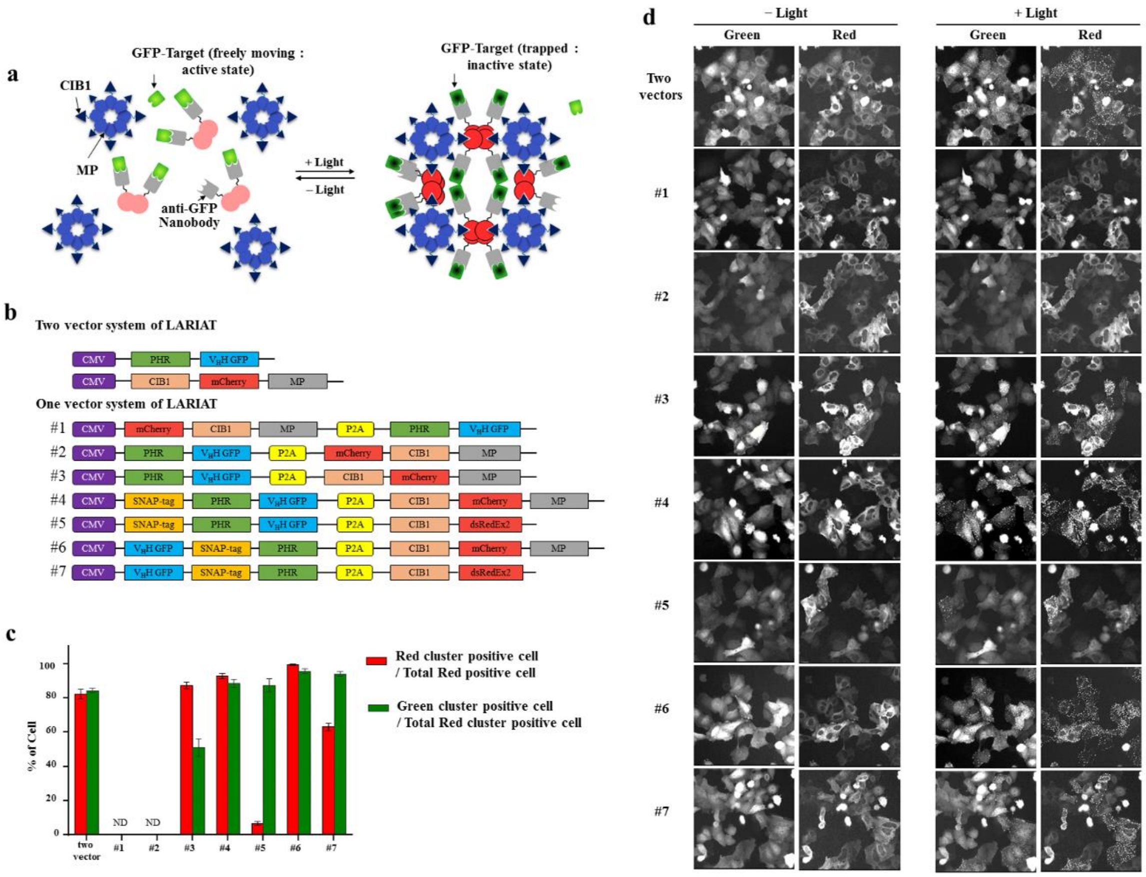

Supplementary Figure 8. Optimization of one vector system for light-activated reversible inhibition by assembled trap (LARIAT).

a. Schematic representation of reversible trapping GFP-labeled proteins by CRY2-conjugated anti-GFP nanobody and CIB1-MP upon light stimulation. **b.** Fusion configuration of one vector LARIAT system using P2A sequence. In order to generate an efficient one vector system, 7 constructs have been designed based on two vector system[1]. **c.** Comparing efficiency of 7 designed constructs in cluster formation. HeLa cells co-expressing GFP with each constructs were illuminated with blue light for 5 minutes at 10 second interval. Cluster formations were analyzed by initial and final frame images. Experiments were repeated twice with multiple wells. Error bars indicate ±s.d. **d.** Representative fluorescence images showing transfected HeLa cells with green GFP and 7 designed constructs before and after light illumination. Images were taken with a 20x objective under solid state light source.

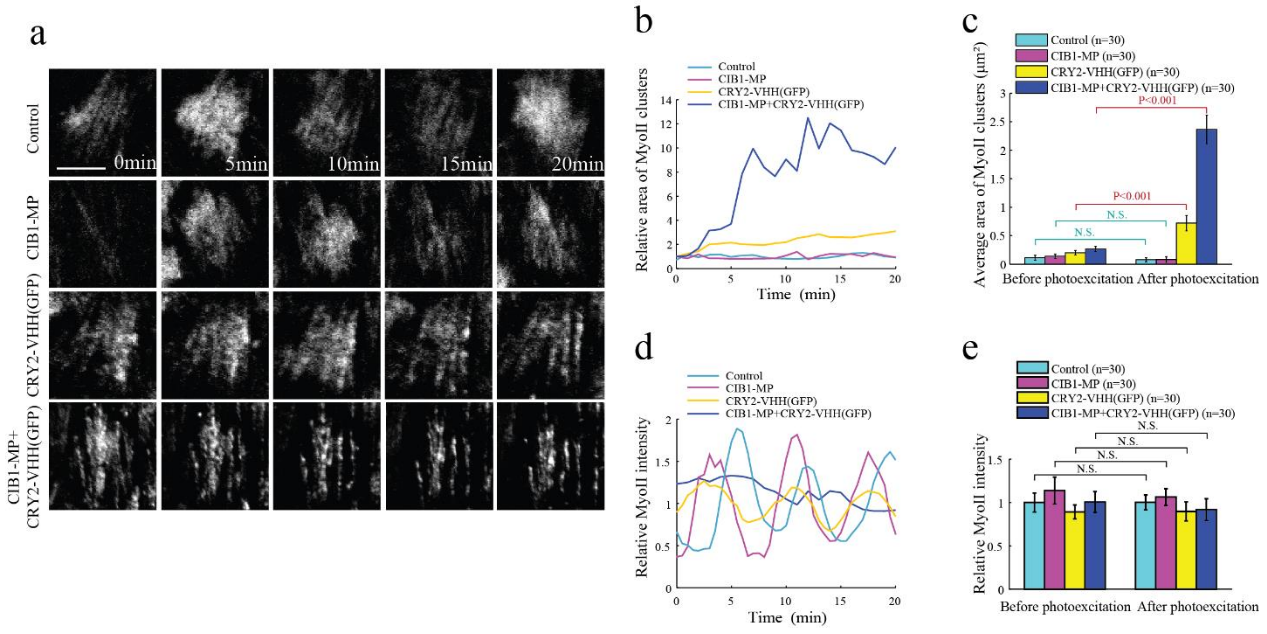

Supplementary Figure 9. Confirmation of the blue light-induced MyoII-GFP cluster formation.

a. Time-lapse series of the representative MyoII-GFP signals in the wild type, CIB1-MP-expressing, CRY2-VHH(GFP)-expressing, and CIB1-MP and CRY2-VHH(GFP)-coexpressing (LARIAT) follicle cells, with the illumination of blue light for 20 minutes at 30 second interval. Scale bar is 5 μm. **b.** Quantification of the dynamic changes of relative MyoII-GFP clustering area in one representative follicle cell with the indicated conditions. **c.** Quantification of average MyoII-GFP clustering area before and after photoexcitation in the indicated conditions. **d.** Quantification of the dynamic changes of relative MyoII-GFP intensity in one representative follicle cell with the indicated conditions. **e.** Quantification of relative MyoII intensity before and after photoexcitation in the indicated conditions. n is the number of samples analyzed. Error bars indicate ±s.d. N.S. means no significant difference, while P<0.001 means significant difference by student’s *t*-test.

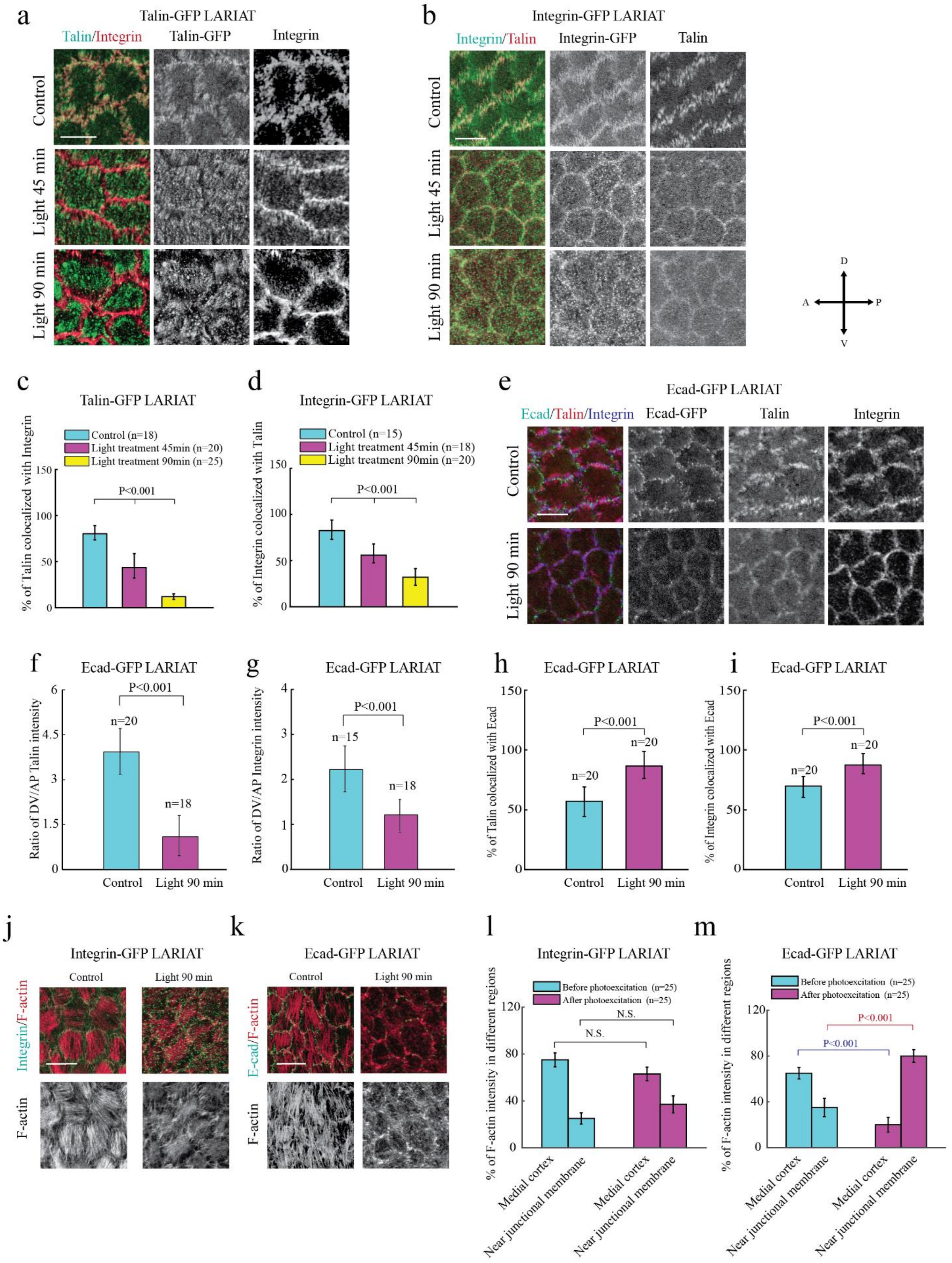
a
Talin-GFP LARIAT
Talin/Integrin
Talin-GFP
Integrin
Control
Light 45 min
Light 90 min
b
Integrin-GFP LARIAT
Integrin/Talin
Integrin-GFP
Talin
D
A
P
V
c
Talin-GFP LARIAT
Control (n=18)
Light treatment 45min (n=20)
Light treatment 90min (n=25)
% of Talin colocalized with Integrin
P<0.001
d
Integrin-GFP LARIAT
Control (n=15)
Light treatment 45min (n=18)
Light treatment 90min (n=20)
% of Integrin colocalized with Talin
P<0.001
e
Ecad-GFP LARIAT
Ecad/Talin/Integrin
Ecad-GFP
Talin
Integrin
f
Ecad-GFP LARIAT
Ratio of DV/AP Talin intensity
P<0.001
n=20
n=18
Control
Light 90 min
g
Ecad-GFP LARIAT
Ratio of DV/AP Integrin intensity
P<0.001
n=15
n=18
h
Ecad-GFP LARIAT
% of Talin colocalized with Ecad
i
Ecad-GFP LARIAT
% of Integrin colocalized with Ecad
j
Integrin-GFP LARIAT
Integrin/F-actin
F-actin
k
Ecad-GFP LARIAT
E-cad/F-actin
l
Integrin-GFP LARIAT
Before photoexcitation (n=25)
After photoexcitation (n=25)
% of F-actin intensity in different regions
N.S.
Medial cortex
Near junctional membrane
m
Ecad-GFP LARIAT
P<0.001

Supplementary Figure 10. Blue light-induced GFP clustering strongly affects cell-matrix adhesion and cell-cell adhesion.

a. Basal view of Talin-GFP and β-Integrin signal marked by antibody staining in the LARIAT-expressing follicle cells with or without light illumination as indicated. **b.** Basal view of β-Integrin-GFP and Talin-mCherry in the LARIAT-expressing follicle cells with or without light illumination as indicated. **c.** Quantification of the percentage of Talin-GFP colocalized with endogenous β-Integrin signal in the indicated conditions. **d.** Quantification of the percentage of β-Integrin-GFP colocalized with Talin-mCherry in the indicated conditions. **e.** Basal view of E-cadherin-GFP, Talin-mCherry and β-Integrin signal marked by antibody staining in the LARIAT-expressing follicle cells with or without light illumination as indicated. **f, g.** Ratio of the DV/AP Talin (**f**) and β-Integrin (**g**) intensities in the indicated conditions. **h, i.** Quantifications of the percentage of Talin-mCherry colocalized with E-cadherin-GFP (**h**) and β-Integrin signal colocalized with E-cadherin-GFP (**i**) in the indicated conditions. **j, k.** Basal views of Integrin-GFP (**j**) and E-cadherin-GFP (**k**), together with F-actin signal marked by phalloidin staining in the LARIAT-expressing follicle cells with or without light illumination as indicated. All scale bars are 10 μm. **l, m.** Quantifications of the relative percentage of F-actin intensity in different subcellular regions as indicated before and after photoexcitation in the conditions with or without LARIAT expression. n is the number of samples analyzed. Error bars indicate ±s.d. N.S. means no significant difference, while $P<0.001$ means significant difference by student's t-test.

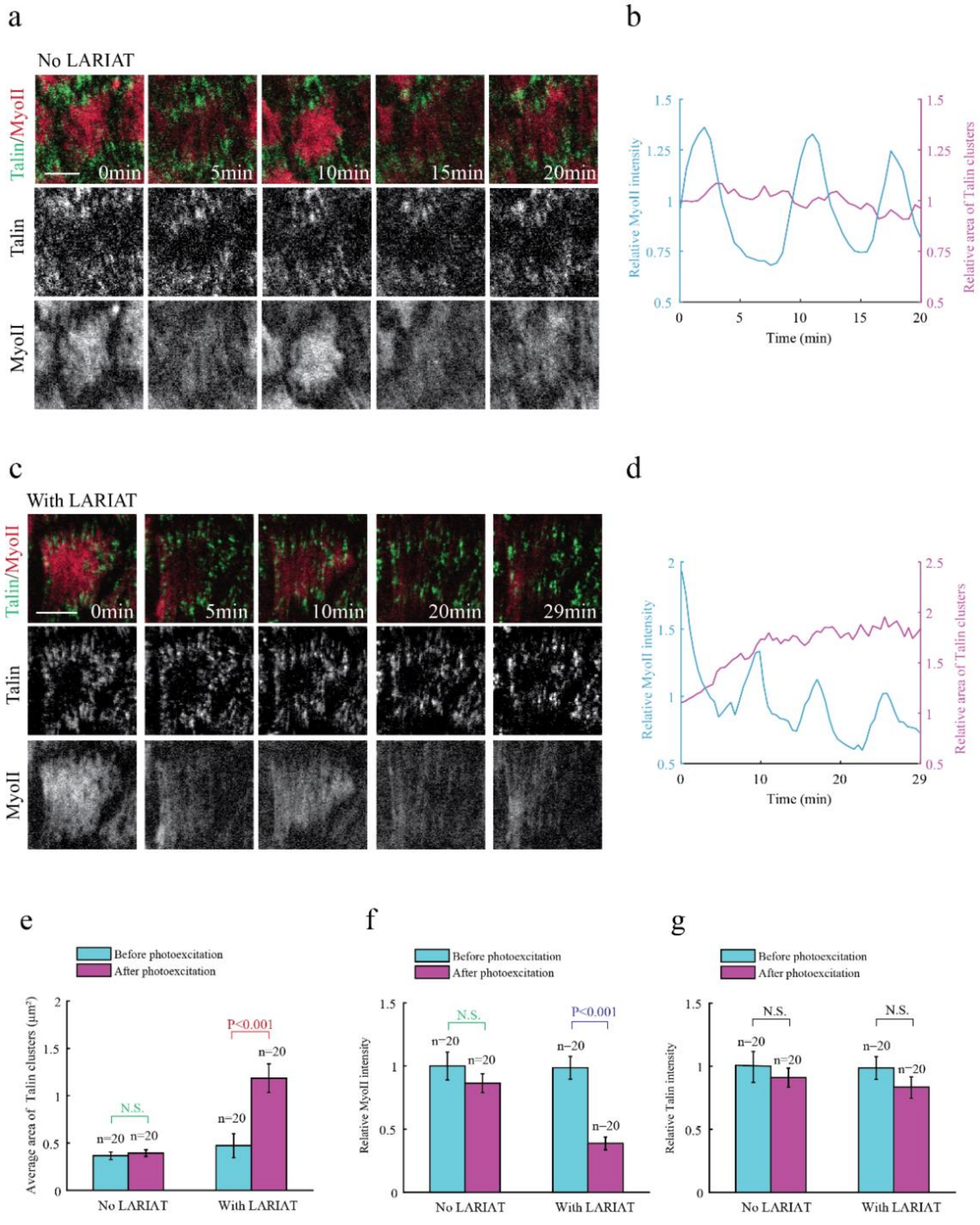

Supplementary Figure 11. Basal MyoII oscillation is inhibited by the light-induced Talin-GFP clustering.

a, c. Time-lapse series of one representative wild type (**a**) and LARIAT-expressing (**c**) follicle cell, labelled with Talin-GFP (here is *Talin-GFP*/+ genotype with one allele of no-GFP-tagged wild type *Talin*) and MyoII-mCherry, and illuminated with blue light for 20-30 minutes at 30

second interval. Both scale bars are 5 μm. **b, d.** Quantifications of the dynamic changes of relative MyoII intensity and relative area of Talin clusters in no LARIAT (**b**) and with LARIAT (**d**) conditions. **e, f, g.** Quantifications of average Talin clustering area (**e**), relative MyoII intensity (**f**) and relative Talin intensity (**g**) before and after photoexcitation in the conditions with or without LARIAT expression. n is the number of samples analyzed. Error bars indicate ±s.d. N.S. means no significant difference, while $P<0.001$ means significant difference by student's *t*-test.

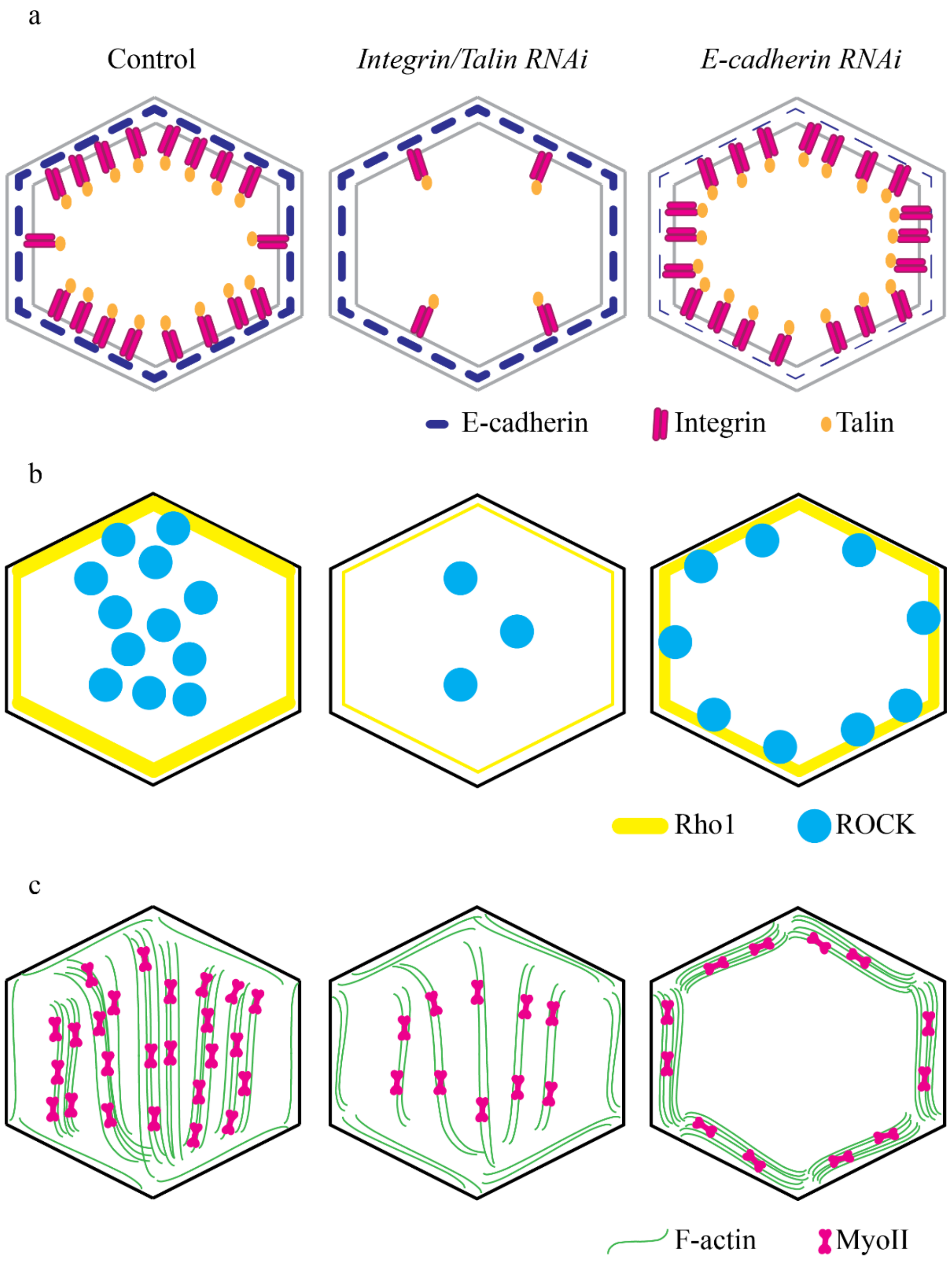

Supplementary Figure 12. Model of basal actomyosin accumulation controlled by cell-matrix adhesion and cell-cell adhesion.

a-c. Schematic representation of the distributions and pattern changes of cell-matrix adhesion and cell-cell adhesion (**a**), Rho1 and ROCK (**b**), and F-actin and MyoII (**c**) at basal domain of individual follicle cell with the indicated genetic backgrounds.

Supplementary Table 1. Primers used to generate amplicons for In-fusion cloning.

Clone No.	Backbone/ Enzyme site	Primer name	Primer sequence (5' to 3')
#1	pmCherry-C1/ EcoRI	CIB1-F	CTCAAGCTTCGAATTCCATGAATGGAGCTATAGGAGGTGAC
		MP-P2A-R	TTGGTGGCATGGGGCAGGACGGAG
		P2A-PHR-F	GCCCCATGCCACCAACTTCAGCCTGCTGAAGCAGGCCGGCGACGTGGAGGAGAACCCCGGCCCCATGAAGATGGACAAAAAGACCATCGTC
		vhhGFP-R	GTCGACTGCAGAATTCTTAGCTGGAGACGGTGACCTG
#2	pEGFP-C1/ AgeI/EcoRI	PHR-F	CGCTAGCGCTACCGGTACCATGAAGATGGACAAAAAGACCATCGTC
		vhhGFP-R	CTGCTTGCTTTAACAGAGAGAAGTTCGTGGCGCCGCTGCCGCTGCTGGAGACGGTGACCTGGG
		P2A-mCherry-F	TGTTAAAGCAAGCAGGAGACGTGGAAGAAAACCCCGGTCCTATGGTGAGCAAGGGCGAGG
		AD-R	GTCGACTGCAGAATTCTCAATGGGGCAGGACGGAGG
#3	pEGFP-C1/ AgeI/EcoRI	PHR-F	CGCTAGCGCTACCGGTACCATGAAGATGGACAAAAAGACCATCGTC
		vhhGFP-P2A-R	CCTGCTTGCTTTAACAGAGAGAAGTTCGTGGCGCCGCTGCCGCTGGAGACGGTGACCTGGG
		P2A-CIB1-F	GTTAAAGCAAGCAGGAGACGTGGAAGAAAACCCCGGTCCTATGAATGGAGCTATAGGAGGTGACCTTTTG
		AD-R	GTCGACTGCAGAATTCTCAATGGGGCAGGACGGAGG
#4	pEGFP-C1/ AgeI/EcoRI	SNAP-F	CGCTAGCGCTACCGGTACCATGGACAAAGACTGCGAAATG
		vhhGFP-P2A-R	CCTGCTTGCTTTAACAGAGAGAAGTTCGTGGCGCCGCTGCCGCTGGAGACGGTGACCTGGG
		P2A-CIB1-F	GTTAAAGCAAGCAGGAGACGTGGAAGAAAACCCCGGTCCTATGAATGGAGCTATAGGAGGTGACCTTTTG
		AD-R	GTCGACTGCAGAATTCTCAATGGGGCAGGACGGAGG
#5	pEGFP-C1/ AgeI/EcoRI	SNAP-F	CGCTAGCGCTACCGGTACCATGGACAAAGACTGCGAAATG
		vhhGFP-P2A-R	CCTGCTTGCTTTAACAGAGAGAAGTTCGTGGCGCCGCTGCCGCTGGAGACGGTGACCTGGG
		P2A-CIB1-F	GTTAAAGCAAGCAGGAGACGTGGAAGAAAACCCCGGTCCTATGAATGGAGCTATAGGAGGTGACCTTTTG
		dsRedEx2-R	GTCGACTGCAGAATTCCTACAGGAACAGGTGGTGGCGGC
#6	pEGFP-C1/ AgeI/EcoRI	vhhGFP-F	CGCTAGCGCTACCGGTGCCACCATGGATCAAGTCCAACTGGTGGAG
		SNAP-vhhGFP-R	TTGTCCATGCCGCTGCCGCTGGAGACGGTGACCTGG
		vhhGFP-	CAGCGGCATGGACAAAGACTGCGAAATGAAGC

		SNAP-F	
		P2A-PHR-R	CCTGCTTGCTTTAACAGAGAGAAGTTCGTGGCGCCGCTGCCGGCAGCACCGATCATAATCTGCG
		P2A-CIB1-F	GTTAAAGCAAGCAGGAGACGTGGAAGAAAACCCCGGTCCTATGAATGGAGCTATAGGAGGTGACCTTTTG
		AD-R	GTCGACTGCAGAATTCTCAATGGGGCAGGACGGAGG
#7	pEGFP-C1/AgeI/EcoRI	vhhGFP-F	CGCTAGCGCTACCGGTGCCACCATGGATCAAGTCCAACTGGTGGAG
		SNAP-vhhGFP-R	TTGTCCATGCCGCTGCCGCTGGAGACGGTGACCTGG
		vhhGFP-SNAP-F	CAGCGGCATGGACAAAGACTGCGAAATGAAGC
		P2A-PHR-R	CCTGCTTGCTTTAACAGAGAGAAGTTCGTGGCGCCGCTGCCGGCAGCACCGATCATAATCTGCG
		P2A-CIB1-F	GTTAAAGCAAGCAGGAGACGTGGAAGAAAACCCCGGTCCTATGAATGGAGCTATAGGAGGTGACCTTTTG
		dsRedEx2-R	GTCGACTGCAGAATTCCTACAGGAACAGGTGGTGGCGGC
#8	pUASt/KpnI	vhhGFP-F	CGGCCGCGCTCGAGGGTACCGCCACCATGGATCAAGTCCAAC
		AD-R	AAAGATCCTCTAGAGGTACCTCAATGGGGCAGGACGGA
#9	pUASt/KpnI	vhhGFP-F	CGGCCGCGCTCGAGGGTACCGCCACCATGGATCAAGTCCAAC
		P2A-CIB1-R	ACCACCACCTCCACCGGTGGCGACCGGTACATGAAT
		AD-F	GGTGGAGGTGGTGGTGGAGGGAAGAGTGGAGGAAAC
		AD-R	AAAGATCCTCTAGAGGTACCTCAATGGGGCAGGACGGA
#10	pUASt/KpnI	vhhGFP-F	CGGCCGCGCTCGAGGGTACCGCCACCATGGATCAAGTCCAAC
		PHR-P2A-R	AAAGATCCTCTAGAGGTACCTCAGAAGTTCGTGGCGCCG
#11	pUASt/KpnI	CIB1-F	CGGCCGCGCTCGAGGGTACCACCATGAATGGAGCTATAGGAGGTG
		AD-R	AAAGATCCTCTAGAGGTACCTCAATGGGGCAGGACGGA

Supplementary Reference

1. Lee, S. *et al.* Reversible protein inactivation by optogenetic trapping in cells. *Nat Methods* **11**, 633-636 (2014).
